# Obesity exacerbates colitis-associated cancer via IL-6-regulated macrophage polarisation and CCL-20/CCR-6-mediated lymphocyte recruitment

**DOI:** 10.1038/s41467-018-03773-0

**Published:** 2018-04-25

**Authors:** Claudia M. Wunderlich, P. Justus Ackermann, Anna Lena Ostermann, Petra Adams-Quack, Merly C. Vogt, My-Ly Tran, Alexei Nikolajev, Ari Waisman, Christoph Garbers, Sebastian Theurich, Jan Mauer, Nadine Hövelmeyer, F. Thomas Wunderlich

**Affiliations:** 1 Max Planck Institute for Metabolism Research Cologne, Institute for Genetics, University of Cologne, Cologne Excellence Cluster on Cellular Stress Responses in Aging-associated Diseases (CECAD), Center for Molecular Medicine Cologne (CMMC), Center for Endocrinology, Diabetes and Preventive Medicine (CEDP) Cologne, 50931 Cologne, Germany; 2 Institute for Molecular Medicine, University Hospital Mainz, 55131 Mainz, Germany; 3 Department of Biochemistry, Kiel University, Medical Faculty, 24118 Kiel, Germany; 4 Department of Pharmacology, Weill Cornell Medical College, Cornell University, New York, NY 10065 USA

## Abstract

Colorectal cancer (CRC) is one of the most lethal cancers worldwide in which the vast majority of cases exhibit little genetic risk but are associated with a sedentary lifestyle and obesity. Although the mechanisms underlying CRC and colitis-associated colorectal cancer (CAC) remain unclear, we hypothesised that obesity-induced inflammation predisposes to CAC development. Here, we show that diet-induced obesity accelerates chemically-induced CAC in mice via increased inflammation and immune cell recruitment. Obesity-induced interleukin-6 (IL-6) shifts macrophage polarisation towards tumour-promoting macrophages that produce the chemokine CC-chemokine-ligand-20 (CCL-20) in the CAC microenvironment. CCL-20 promotes CAC progression by recruiting CC-chemokine-receptor-6 (CCR-6)-expressing B cells and γδ T cells via chemotaxis. Compromised cell recruitment as well as inhibition of B and γδ T cells protects against CAC progression. Collectively, our data reveal a function for IL-6 in the CAC microenvironment via lymphocyte recruitment through the CCL-20/CCR-6 axis, thereby implicating a potential therapeutic intervention for human patients.

## Abstract (web summary)

Inflammation can be induced by obesity, and has been linked with onset of colorectal cancer (CAC). Here the authors show in mouse models that obesity-induced interleukin-6 alters macrophage function to enhance CCL-20/CCR-6-mediated recruitment of B cells and γδ T cells, thereby promoting gut inflammation and CAC progression.

## Introduction

The current obesity epidemic not only accounts for the increased incidence of classical comorbidities such as type 2 diabetes mellitus, but also predisposes to the development of certain cancers—primarily those that require an inflammatory tumour microenvironment (TME)^1^. One cancer type that is strongly associated with obesity is colorectal cancer (CRC)^2–4^. Globally, CRC is the second most diagnosed cancer in females and the third in males with 14.1 million new cancer cases and 8.2 million deaths in 2012^5^.

Obesity-induced alterations in microbiota composition and stem cell modulation have been demonstrated to promote CRC development^6,7^, but therapeutic strategies targeting these putative drivers of CRC might have unpredictable side effects. It is well-established that obesity is associated with a chronic, low-grade inflammatory state^8^ that could also contribute to CRC development. However, the role of obesity-induced inflammation in CRC development is unknown. Importantly, obesity therapeutic strategies that reduce inflammation can be easily conducted in patients via dietary and lifestyle intervention^9^. Thus, reducing obesity-associated inflammation might represent a convenient strategy to prevent obesity-induced CRC.

In obesity, immune cells such as macrophages, T cells and B cells infiltrate the white adipose tissue. Activation of these cells causes local and systemic increases of inflammatory cytokines, such as tumour necrosis factor (TNF) and interleukin (IL)-6. Elevated cytokine levels are typically associated with obesity and propagate the obesity-associated inflammatory state^10–13^.

IL-6 acts via its membrane-bound IL-6 receptor (IL-6R) composed of IL-6Rα that mediates specificity and the common signalling chain of IL-6-type cytokines glycoprotein 130 (GP130)^14^. Though previously excluded, also ciliary neurotrophic factor (CNTF), another IL-6-type cytokine, can act as an alternative ligand for the IL-6R under certain circumstances, which might explain different outcomes when investigating IL-6 and IL-6R knockout mice^15^. Moreover, cell types that are not expressing IL-6Rα can be rendered IL-6-sensitive via IL-6 transsignalling mechanisms where a soluble IL-6Rα (sIL-6Rα) is shedded from the cell surface and acts with IL-6 on GP130-expressing cells^16^. Interestingly, such IL-6 transsignalling prevents obesity-induced recruitment of macrophages into adipose tissue that paradoxically failed to improve systemic insulin sensitivity^17^. On the other hand, enhanced central sIL-6Rα signalling improved energy and glucose homoeostasis in obesity^18^. Thus, different modes of signalling can affect various cell types that even do not express the necessary receptors. Moreover, we have demonstrated previously that IL-6 exerts beneficial effects in lean mice by limiting hepatic inflammation, whereas the chronic low-grade elevation of IL-6 in obesity abrogates these functions, presumably via the development of IL-6 resistance^19–22^. Moreover, IL-6 signalling can polarise macrophages towards an anti-inflammatory M2 phenotype, whereas IL-6Rα deficiency leads to largely arrested macrophages in the proinflammatory M1 state^19^. Notably, M2 macrophages functionally overlap with tumour-associated macrophages, indicating that IL-6 might have a detrimental role in carcinogenesis^23,24^.

Indeed, IL-6 promotes CAC development via its action in intestinal epithelial cells (IEC)^25–28^. Furthermore, in the classical aetiology of CAC, the initial development of inflammatory bowel diseases (IBD) such as colitis ulcerosa and Crohn’s disease are also associated with increased IL-6 level in circulation^29^. This suggests that induction of IL-6 could be a common mechanism shared between obesity-induced and IBD-induced disease progression. However, how the low-grade nature of IL-6 in obesity impacts on CRC development and progression has not been investigated yet.

Here we investigate the role of obesity-induced IL-6 during development and progression of CAC in mice. We demonstrate that macrophage-specific IL-6Rα inactivation strongly ameliorates CAC in obesity. This is owing to a reduction of the chemoattractant CC-chemokine-ligand-20 (CCL-20) derived from M2 macrophages, which in turn facilitates recruitment of B cells and γδ T cells into the TME in a CC-chemokine-receptor-6 (CCR-6) dependent manner. Thus, we identify IL-6R signalling in macrophages as an important mediator of colon carcinogenesis during obesity.

## Results

### Diet-induced obesity increases CAC development

In a first experiment, we aimed at elucidating whether diet-induced obesity affects colon inflammation and CAC. To model obesity-induced CAC in mice, we exposed cohorts of C57BL/6 mice to either normal chow (NCD) or high-fat diet (HFD) feeding from weaning on. As expected, 8-week-old HFD-fed animals exhibited increased body weight and body fat content with increased serum insulin and leptin levels as well as impaired glucose homoeostasis (Supplementary Fig. 1a-e).

**Fig. 1:**
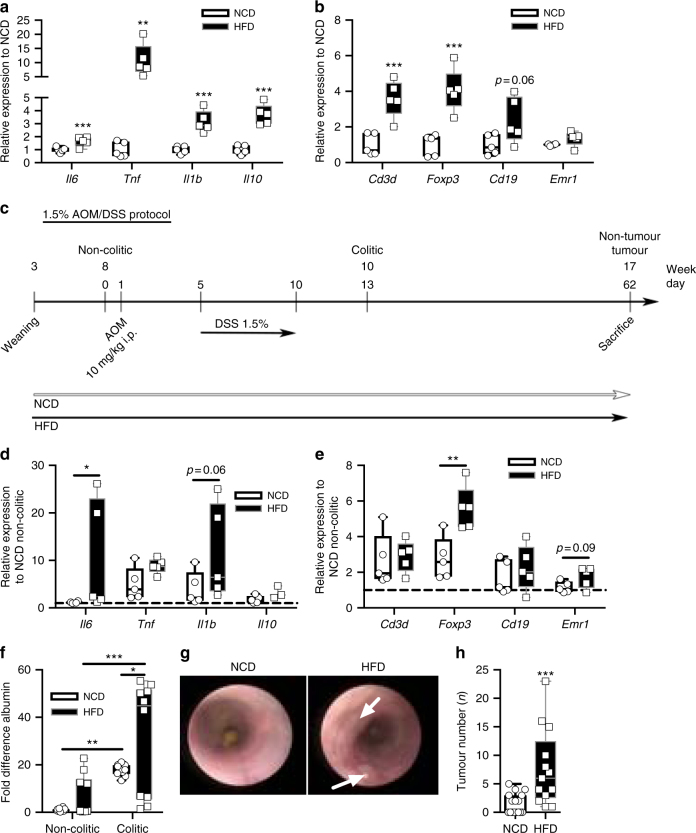
Obesity enhances colonic inflammation and CAC tumour burden in C57BL/6 mice. **a**, **b** qPCR analysis of indicated gene expression in distal colons derived from 8-week-old non-colitic NCD (*n* = 5) and HFD-fed (*n* = 5) C57BL/6 mice; following results are presented relative to non-colitic NCD. **c** Scheme of the 1.5% AOM/DSS protocol of mice fed a NCD or a HFD from weaning (3 weeks of age) until the end of the experiment with 17 weeks of age. 8-week-old mice were injected with 10 mg/kg AOM i.p. at day 1 of the 1.5% AOM/DSS protocol. From day 5 on, mice received 1.5% DSS in the drinking water for 5 days. Mice were either killed with 8 weeks of age at day 0 (non-colitic), 10 weeks of age at day 13 for analysis of colitic conditions (colitic) or with 17 weeks of age at day 62 (non-tumour and tumour) for analysis of tumourigenesis. **d**, **e** qPCR analysis of indicated gene expression in distal colons of 10-week-old NCD (*n* = 5) and HFD-fed (*n* = 5) colitic C57BL/6 mice; results are presented relative to non-colitic NCD. **f** Normalised albumin concentration in faeces of NCD (*n* = 10) and HFD-fed (*n* = 10) C57BL/6 mice under non-colitic (day 0) and colitic (day 13) conditions. **g** Representative endoscopic pictures of 13-week-old NCD and HFD-fed C57BL/6 mice of the 1.5% AOM/DSS protocol. **h** Tumour number of 17-week-old NCD (*n* = 18) and HFD-fed (*n* = 13) C57BL/6 mice counted at day 62 of the 1.5% AOM/DSS protocol. AOM, azoxymethane; DSS, dextran sodium sulphate, NCD, normal chow diet; HFD, high-fat diet; CAC, colitis-associated colorectal cancer. centre line: median; box limits: 1st and 3rd quartiles; whisker: maximum to minimum, *p ≤ 0.05, **p ≤ 0.01 and ***p ≤ 0.001 two-tailed unpaired Student’s *t*-test **a**, **b**, **h** or two-way ANOVA followed by Fisher LSD **d**–**f**

Next, we investigated expression of inflammatory cytokines as well as markers for immune cells in the colon tissue of these mice. This analysis revealed the elevated expression of inflammatory cytokines *Il6*, *Tnf*, *Il1β* and *Il10* in the colon of obese mice compared with lean controls (Fig. 1a). Examination of immune cell markers in the obese colon revealed increases in T cells (*Cd3δ*), regulatory T cells (Tregs) (forkhead-box-protein P3 (*Foxp3)*), B cells (*Cd19*) but similar expression of the common macrophage marker epidermal growth factor-like module-containing mucin-like hormone receptor-like 1 (*Emr1*) (Fig. 1b). Thus, HFD-feeding accelerates inflammation and lymphocytes in the colon of mice.

Next, we subjected cohorts of NCD- and HFD-fed mice to the established colitis-associated adenoma protocol induced by azoxymethane (AOM) injection directly followed by three repetitive cycles of 2.5% dextrane sodium sulphate (DSS) in the drinking water over 7 days (Supplementary Fig. 1f)^30^. Strikingly, obese mice either died spontaneously or had to be killed during the first DSS cycle, as these animals lost more than 20% of their initial body weight owing to symptoms associated with exaggerated colitis (Supplementary Fig. 1g, h). As the standard CAC protocol turned out to be highly lethal for obese mice, we adjusted the conditions accordingly to ensure survival of all cohorts throughout the treatment period. Therefore, we injected 8-week-old mice with AOM (day 1) and supplemented the drinking water with 1.5% DSS from day 5 for 5 days. At day 10 of the 1.5% AOM/DSS protocol the animals received normal water until the end of the experiment at day 62 (non-tumour, tumour) (Fig. 1c). To examine the colitis phase animals were killed with 10 weeks of age at day 13 (colitic) and as control with 8 weeks of age at day 0 (non-colitic) of the 1.5% AOM/DSS protocol.

Notably, lean mice that developed colitis exhibited an adapted colonic microenvironment at day 13 that resembled the inflammatory state of non-colitic HFD-fed mice (Fig. 1a, b, d, e). Strikingly, HFD in colitic mice increased *Il6* expression and to a lower extent *Tnf*, *Il1β* and *Il10* (Fig. 1d). Although colitic mice, independent of diet, had similar presence of T cells and B cells in colons compared with non-colitic HFD-fed colons, expression of the Treg transcription factor FoxP3 was increased suggesting a shift in T-cell composition (Fig. 1b, e). Macrophages increased only in tendency in obese colitic colons (Fig. 1e). Thus, HFD feeding mimics the inflammatory phenotype found in colitis.

Colitis compromises the gut barrier function, reflected by rectal bleeding, by mounting immune responses against commensal factors such as bacterial lipopolysaccharides (LPS)^30^. To examine gut barrier function and rectal bleeding, we measured the presence of blood-abundant albumin in faeces of mice^31^. This analysis showed elevated immunoreactive albumin in the faeces of HFD-fed mice both under non-colitic and colitic conditions, indicating impaired gut barrier function (Fig. 1f). Further colonic investigation using endoscopy^32^ revealed elevated presence of dysplastic neoplasia in colitic HFD mice compared with NCD-fed mice (Fig. 1g). Importantly, macroscopic investigation of colons demonstrated an increased tumour burden in obese mice when compared with their lean littermates at day 62 (Fig. 1h and Supplementary Fig. 1i). Furthermore, colons of diet-induced obese mice were shortened, which is indicative of elevated inflammation and colitis (Supplementary Fig. 1j)^30^. Moreover, we observed an exaggerated immune cell infiltration into tumours of obese mice as determined by histochemistry using F4/80 and CD3 antibodies to identify macrophages and T cells (Supplementary Fig. 1k, l). Taken together, diet-induced obesity exaggerated colonic inflammation, impaired gut barrier function and enhanced CAC development. Furthermore, HFD-feeding *per se* induced colonic inflammation, and colitis in lean mice increased inflammation to levels of non-colitic HFD-fed mice. Importantly, of the investigated cytokines, only IL-6 was upregulated in colitis of obese mice implicating that IL-6 might have a role in obesity-induced CAC development.

### IL-6Rα deficiency in myeloid cells reduces CAC development

**Fig. 2:**
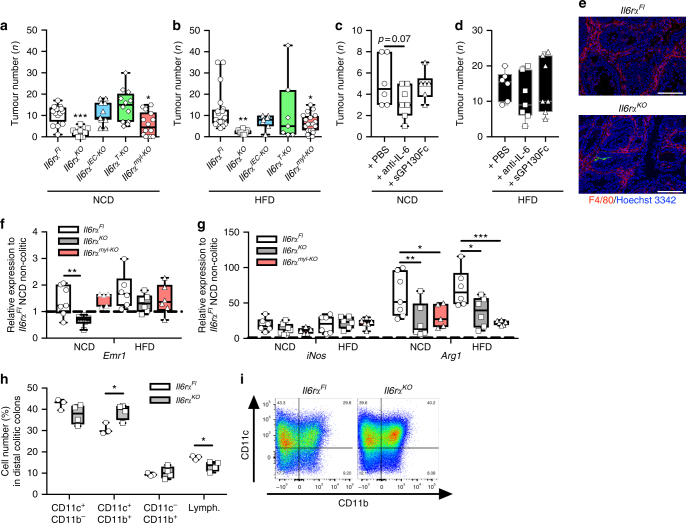
IL-6Rα deficiency in myeloid cells reduces CAC. AOM/DSS-induced tumour numbers in colons of **a** NCD-fed (*n* = 9–16) and **b** HFD-fed (*n* = 7–20) 17-week-old *Il6r*α^*Fl*^, *Il6r*α^*KO*^, *Il6r*α^*IEC-KO*^, *Il6r*α^*T-KO*^, *Il6r*α^*myl-KO*^ mice at day 62 of the 1.5% AOM/DSS protocol. Tumour numbers counted at day 62 of 17-week-old **c** NCD (*n* = 6–7) or **d** HFD (*n* = 6–7)-fed C57BL/6 mice injected with either PBS, 500 µg anti-IL-6 antibody or 150 μg sGP130Fc at day 3 of the 1.5% AOM/DSS protocol. **e** Representative immunofluorescent staining of macrophages using F4/80 antibody (red) counterstained by Hoechst 3342 (blue) in colons of 17-week-old HFD-fed *Il6r*α^*Fl*^ and *Il6r*α^*KO*^ mice at day 62 of the 1.5% AOM/DSS protocol. **f**, **g** qPCR analysis of indicated gene expression in tumours (*n* = 6–7) of 17-week-old NCD or HFD-fed *Il6r*α^*Fl*^, *Il6r*α^*KO*^ and *Il6r*α^*myl-KO*^ mice at day 62 of the 1.5% AOM/DSS protocol, results are presented relative to non-colitic distal colon of *Il6r*α^*Fl*^ NCD at day 0. **h** Relative cell number (%) of CD11c^+^/ CD11b^−^ dendritic cells, CD11c^+^/CD11b^+^ and CD11c^−^/CD11b^+^ macrophages and CD11c^−^/CD11b^−^ lymphocytes from lymphocyte gate SSC/FSC in colitic colons of NCD-fed *Il6r*α^*Fl*^ and *Il6r*α^*KO*^ mice at day 13 of the 1.5% AOM/DSS protocol. **i** Representative FACS plots of CD11c and CD11b expression in immune cells from **h**. AOM, azoxymethane; DSS, dextran sodium sulphate; NCD, normal chow diet; HFD, high-fat diet; CAC, colitis-associated colorectal cancer. Centre line: median; box limits: 1st and 3rd quartiles; whisker: maximum to minimum, *p ≤ 0.05, **p ≤ 0.01 and ***p ≤ 0.001 two-tailed unpaired Student’s *t*-test **h**, one-way **a**–**d** or two-way ANOVA followed by Fisher LSD **f**,** g**. Scale bar, 100 μm

Next, we asked whether the obesity-induced elevation in IL-6 is driving CAC development. First, to identify the cell type that responds to IL-6 we induced CAC in lean and obese mice with conditional ablation of the IL-6Rα in all cells^33^ (*Il6rα*^*KO*^), in IECs (*Il6rα*^*IEC-KO*^)^27^, in mature T cells^34^ (*Il6rα*^*T-KO*^) or in the myeloid lineage^19^ (*Il6rα*^*myl-KO*^) using the 1.5% AOM/DSS protocol (Fig. 1c). As a consequence of Cre recombinase expression, exons 2 and 3 of the *Il6rα*^*FL*^ allele are excised to conditionally inactivate IL-6Rα in the respective cell type (Supplementary Fig. 2a-d). In line with a previous report employing IL-6 knockout mice, IL-6Rα deficiency protected against CAC development under lean and under obese conditions (Fig. 2a, b, Supplementary Fig. 2e–j)^25^. The cell type-specific inactivation of IL-6 signalling revealed that IEC-specific and T cell-specific inactivation of IL-6Rα did not decrease CAC development. However, myeloid lineage-specific IL-6Rα deficiency protected against CAC (Fig. 2a, b, Supplementary Fig. 2e-j). Macrophages can provide IL-6 and sIL-6Rα in CAC, but IL-6 signalling intrinsic in macrophages might also have a role in CAC^25^. To investigate whether neutralising IL-6 or sIL-6Rα affects CAC, we injected anti-IL-6 antibody or the designer cytokine soluble GP130 (sGP130Fc) before the colitis phase at day 3 of the 1.5% AOM/DSS protocol in lean and obese C57BL/6 mice. C57BL/6 mice had similar body weight loss in colitis independent of anti-IL-6 and sGP130Fc treatment under NCD and HFD conditions (Supplementary Fig. 2k, l). Of note, tumour numbers were only reduced in tendency upon neutralisation with anti-IL-6 in lean and obese mice (Fig. 2c, d, Supplementary Fig. 2m-r). On the other hand, lean and obese mice with blockade of sIL-6Rα signalling had similar tumour numbers independent of neutralisation (Fig. 2c, d). However, anti-IL-6 and sGP130Fc treatment in lean mice reduced afflicted colonic area (Supplementary Fig. 2m), which is in line with a previous report, demonstrating IL-6 deficiency reducing tumour area in CAC^25^. Of note, other IL-6-type cytokines such as CNTF that have not been blocked by antibody-mediated IL-6 depletion and blockade of sIL-6Rα via sGP130Fc might compensate in CAC development. Nevertheless, neutralising IL-6 and sIL-6Rα agents are less effective than genetic IL-6Rα ablation and in line with our previous experiments might even suggest macrophage-intrinsic IL-6 signalling functions impacting on CAC.

In this context, we have previously demonstrated that IL-6Rα deficiency in the myeloid compartment impedes M2 macrophage polarisation^19^. Furthermore, reports allocate M2-like macrophages a tumour-promoting function via angiogenic and immunomodulatory factors^35^. On the other hand, M1-like macrophages combat tumours via their proinflammatory and cytotoxic potential^36^. As we observed similar total macrophage numbers in tumour tissues independent of genotype (Fig. 2e, f), we next asked whether IL-6Rα deficiency might alter specific macrophage subsets in CAC. Thus, we examined gene expression to identify M1/*inducible nitric oxide synthase* (*iNos)*- and M2/*arginase* 1 (*Arg1*)-expressing macrophages on isolated tumour tissue derived from control, *Il6rα*^*KO*^ and *Il6rα*^*myl-KO*^ mice. This analysis revealed that IL-6Rα deficiency in macrophages largely prevented M2-like polarisation as evidenced by reduced *Arg1* expression, whereas *iNos* expression and thus M1 polarisation remained unaltered (Fig. 2g). Thus, a compromised M2 polarisation might hamper CAC in IL-6Rα-deficient mice. In line with this finding, fluorescence-activated cell sorting (FACS) analysis of immune cells revealed increases in CD11c^+^ M1 macrophages in colitis colons of *Il6rα*^*KO*^ mice, whereas the lymphocytes were reduced in Il-6rα-deficient colons (Fig. 2h, i). These findings suggest that a shifted macrophage polarisation capacity towards the inflammatory M1 lineage is contributing to the reduced CAC development found in *Il6rα*^*KO*^ and *Il6rα*^*myl -KO*^ mice.

Altered macrophage composition might affect DSS-induced colitis, impairments of gut barrier function and proliferation of IECs by providing growth factors and cytokines. In line with this evidence, increased inflammation and IEC death in a three times DSS-mediated colitis of IL-6-deficient mice is a result of impaired IL-6-mediated signal transducer and activator of transcription 3 (Stat3) action providing survival capacities in normal and premalignant IECs^25^. However, colitis symptoms such as weight loss and faecal albumin were comparable between control and *Il6rα*^*KO*^ mice under NCD and HFD conditions, which might be a consequence of the mild HFD-adapted CAC protocol applied here (Supplementary Fig. 3a–d). Moreover, even colitis-associated proliferation as examined via western blot analyses against the proliferation marker proliferating cell nuclear antigen (PCNA) was similarly detectable in control and IL-6Rα-deficient samples during colitis (Supplementary Fig. 3e, f). In contrast, IL-6Rα-deficient tumours had less PCNA immunoreactivity compared with control tumours, whereas tumours from HFD-fed control mice had increased PCNA levels indicative of increased proliferation compared with NCD-fed controls (Supplementary Fig. 3g, h). Furthermore, decreased proliferation of IL-6Rα-deficient tumours was confirmed via Ki67 staining (Supplementary Fig. 3i, j). Thus, whereas colitis and colitis-associated proliferation is not affected by IL-6Rα deficiency, tumours of *Il6rα*^*KO*^ mice exhibited less PCNA and Ki67 reactivity. Hence, the altered macrophage composition in IL-6Rα-deficient mice is not affecting the colitis phase of CAC but impacts on later stages of tumourigenesis.

### IL-6Rα deficiency attenuates M2-mediated CCL-20 expression

**Fig. 3:**
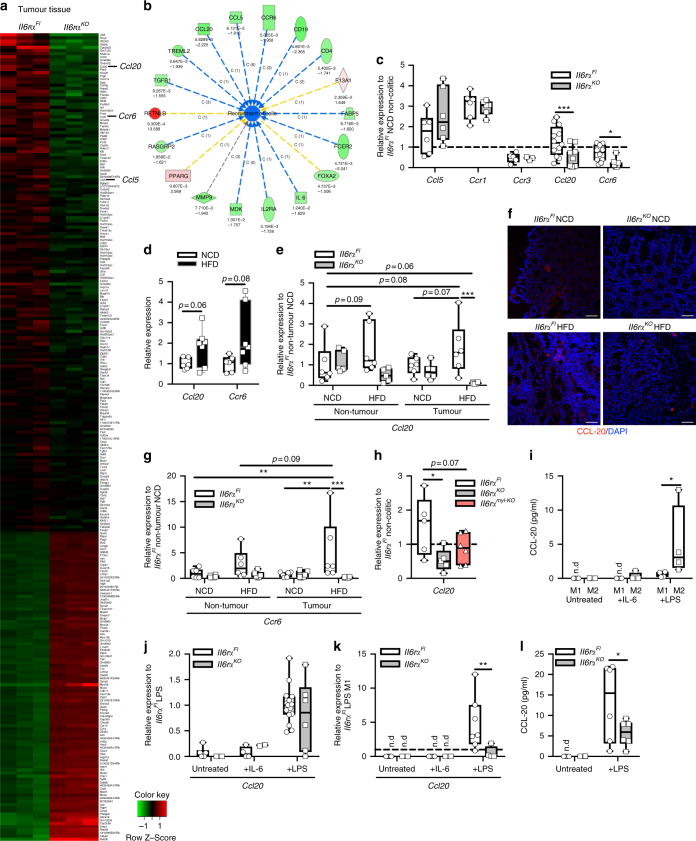
IL-6-polarised M2 macrophages express CCL-20. **a** Heat map of differently expressed transcripts in tumours derived from 17-week-old HFD-fed *Il6r*α^*Fl*^ versus *Il6r*α^*KO*^ mice at day 62 of the 1.5% AOM/DSS protocol, assessed with a cutoff of a change in expression of 1.5-fold and *p*-value ≤ 0.025. **b** IPA analysis diagram of downregulated (green) and upregulated (red) cell recruitment factors in tumour tissues derived from 17-week-old HFD-fed *Il6r*α^*Fl*^ versus *Il6r*α^*KO*^ mice at day 62 of the 1.5% AOM/DSS protocol. Upper number represent respective *p*-value, lower number fold change. **c** qPCR analysis of indicated gene expression in tumours of 17-week-old HFD-fed *Il6r*α^*Fl*^ versus *Il6r*α^*KO*^ (*n* = 6–12) at day 62 of the 1.5% AOM/DSS protocol, results are presented relative to non-colitic NCD *Il6r*α^*Fl*^ colons at day 0. **d** qPCR analysis of indicated gene expression in distal colons of 8-week-old NCD (*n* = 6) and HFD (*n* = 7) fed C57BL/6 mice. **e** qPCR analysis of *Ccl20* expression in non-tumour tissue and tumours of 17-week-old NCD and HFD-fed *Il6r*α^*Fl*^ and *Il6r*α^*KO*^ mice (*n* = 6) at day 62 of the 1.5% AOM/DSS protocol, results are presented relative to NCD *Il6r*α^*Fl*^ non-tumour at day 62. **f** Representative immunofluorescent stainings of CCL-20 (red) expression in colons of 17-week-old NCD and HFD-fed *Il6r*α^*Fl*^ and *Il6r*α^*KO*^ mice at day 62 of the 1.5% AOM/DSS protocol. **g** qPCR analysis of *Ccr6* expression in non-tumour tissue and tumours of 17-week-old NCD and HFD-fed *Il6r*α^*Fl*^ and *Il6r*α^*KO*^ mice (*n* = 6) at day 62 of the 1.5% AOM/DSS protocol, results are presented relative to NCD *Il6r*α^*Fl*^ non-tumour at day 62. **h** qPCR analysis of *Ccl20* gene expression in F4/80 MACS-purified macrophages from colitic colons of 10-week-old NCD-fed *Il6r*α^*Fl*^, *Il6r*α^*KO*^ and *Il6r*α^*myl*-*KO*^ (*n* = 6) at day 13 of the 1.5% AOM/DSS protocol, results are presented relative to macrophages F4/80 MACS sorted from non-colitic *Il6r*α^*Fl*^ colons. **i** CCL-20 produced by M1- and M2-polarised BMDM from control mice (*n* = 4) stimulated 24 h with IL-6 or LPS as examined by ELISA. qPCR analysis of *Ccl20* gene expression in **j** M1 (*n* = 4–15) or **k** M2 (*n* = 7)-polarised BMDM stimulated with IL-6 or LPS for 8 h from *Il6r*α^*Fl*^ and *Il6r*α^*KO*^ mice, results are presented relative to LPS-stimulated *Il6r*α^*Fl*^ M1. **l** CCL-20 produced by M2-polarised BMDM stimulated 36 h with LPS from *Il6r*α^*Fl*^ and *Il6r*α^*KO*^ mice (*n* = 6) as examined by ELISA. AOM, azoxymethane; DSS, dextran sodium sulphate; NCD, normal chow diet; HFD, high-fat diet; IPA, Ingenuity pathway analysis; MACS, magnetic-activated cell sorting; BMDM, bone marrow-derived macrophages; n.d., not determined. centre line: median; box limits: 1st and 3rd quartiles; whisker: maximum to minimum, *p ≤ 0.05, **p ≤ 0.01 and ***p ≤ 0.001 two-tailed unpaired Student’s *t*-test **d**, one-way **h** or two-way ANOVA followed by Fisher LSD **c**, **e**, **g**, **i**, **j**, **k**, **l**. Scale bar, 50 μm

To gain further insights into the molecular mechanisms that promote CAC via obesity-induced IL-6, we compared global gene expression in obese control vs IL-6Rα-deficient tumour samples by microarray. Although examination of upregulated genes failed to clearly identify pathways, a detailed analysis of downregulated genes revealed that the TME has been altered in the absence of IL-6 signalling (Fig. 3a, Supplementary Table 1). Gene Ontology analyses revealed that cell recruitment was impaired in knockout tumours compared with controls. Here, important chemoattractants such as *Ccl20* and *Ccl5* were downregulated in the microarray of IL-6Rα-deficient tumours (Fig. 3b). *Ccl20* and its unique receptor *Ccr6* were among the 30 genes that were downregulated the highest in IL-6Rα-deficient tumours (Fig. 3a, Supplementary Table 1). However, although expression of *Ccl5* and its receptors *Ccr1* and *Ccr3* were not affected by IL-6Rα deficiency in CAC, expression levels of *Ccl20* and of its unique receptor *Ccr6* were reduced in knockout tumours (Fig. 3c). Consistently, *Ccl20* and *Ccr6* were elevated in non-colitic obese mice (Fig. 3d). Moreover, *Ccl20* and *Ccr6* increased in obese control tumours and were downregulated in non-tumour tissue and tumours of obese IL-6Rα-deficient mice (Fig. 3e–g). Thus, we assumed that chemoattraction of cells via the CCL-20/CCR-6 axis might affect CAC, whereas the source for CCL-20 is unclear.

CCL-20/macrophage inflammatory protein-3α derives from macrophages in numerous types of tumours, which causes the recruitment of CCR-6-expressing lymphocytes^37–39^. To this end, we Magnetic-activated cell sorting (MACS)-separated macrophages and IECs from colitic colons derived from control, *Il6rα*^*KO*^ and *Il6rα*^*myl-KO*^ mice and subjected them to qPCR to examine *Ccl20* expression. This analysis revealed increased *Ccl20* expression in control macrophages isolated from colitic colons when compared with IL-6Rα-deficient macrophages (Fig. 3h), whereas IL-6Rα inactivation via LysM-Cre had a tendency towards reduced *Ccl20* expression (Fig. 3h). However, also control IEC samples exhibited increased *Ccl20* expression in colitis either via IEC-derived *Ccl20* expression or potentially via contaminating macrophages in the samples (Supplementary Fig. 4a). From our previous experiments that demonstrate IL-6 signalling in macrophages as crucial mediator in CAC, we speculated that intestinal macrophages are a main source of CCL-20 in CAC. Therefore, IL-6 signalling in macrophages might either directly control CCL-20 expression on the transcriptional level or indirectly via its ability to polarise towards M2 macrophages. To test these hypotheses, we generated bone marrow-derived macrophages (BMDM) from control C57BL/6 mice and polarised them towards M1 via LPS/interferon-γ (IFNγ) treatment or to M2 via IL-4/IL-6 treatment. Examination of CCL-20 in the supernatant revealed that IL-6 stimulation did not directly induce CCL-20 expression in both M1 and M2 (Fig. 3i). However, LPS stimulation mimicking invading commensals increased CCL-20 in M2 compared to LPS-stimulated M1 macrophages (Fig. 3i). This experiment revealed that IL-6 indirectly controls CCL-20 expression in macrophages via its ability to polarise towards M2-type macrophages.

To confirm that IL-6Rα-deficient macrophages fail to produce CCL-20, we subjected control and *Il6rα*^*KO*^ BMDM to M1 and M2 protocols and stimulated them with IL-6 and LPS, respectively. Consistent with our previous finding, IL-6Rα-deficient M2 had a blunted ability to differentiate into M2 macrophages substantiated by a compromised upregulation of M2 markers *Arg1* and *Il4rα* upon IL-6 stimulation (Supplementary Fig. 4b, c). Of note, M1 differentiation was similar between control and IL-6Rα-deficient macrophages as revealed by *iNos* expression (Supplementary Fig. 4d). Whereas IL-6 stimulation of M1 and M2 showed undeterminable *Ccl20* expression, LPS-stimulated control M2 increased *Ccl20* expression compared with the blunted response in IL-6Rα deficiency (Fig. 3j, k). Furthermore, control M2 secreted more CCL-20 compared with IL-6Rα-deficient BMDM in the M2 protocol under LPS-stimulated conditions (Fig. 3l). Thus, CCL-20 expression in macrophages is not directly controlled via IL-6-induced transcriptional regulation but instead, IL-6 polarises macrophages in the TME towards M2 capable to produce CCL-20 upon exposure to commensal antigens such as LPS.

Collectively, these experiments suggest that CCL-20 is expressed by M2-type macrophages in colitis and that the inability of IL-6Rα-deficient macrophages to polarise towards M2-type impedes on CCL-20 expression and presumably on recruitment of CCR-6-expressing cells. Thus, the CCL-20/CCR-6 axis might recruit cell types that promote CAC development.

### IL-6Rα-deficient tumours have reduced lymphocyte quantity

**Fig. 4:**
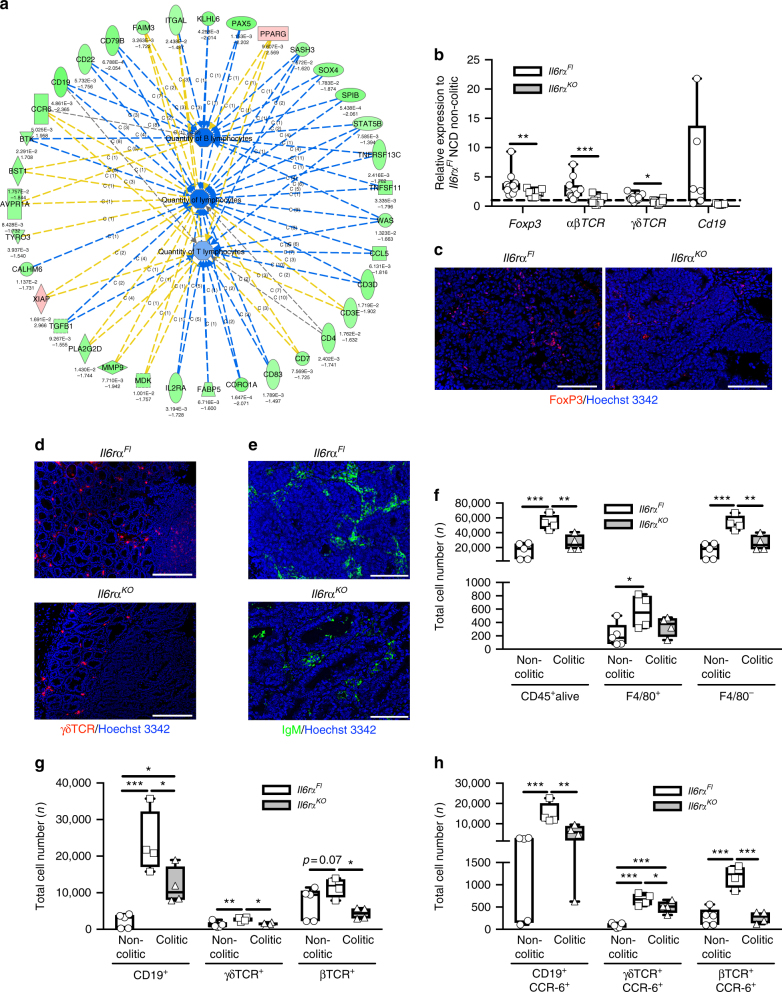
IL-6Rα deficiency compromises CCR-6-expressing lymphocyte recruitment in CAC. **a** IPA analysis diagram of quantity of downregulated (green) and upregulated (red) lymphocyte-specific genes in tumours derived from 17-week-old HFD-fed *Il6r*α^*Fl*^ versus *Il6r*α^*KO*^ mice at day 62 of the 1.5% AOM/DSS protocol. Upper number represent respective *p*-value, lower number fold change. **b** qPCR analysis of indicated gene expression in tumours of 17-week-old *Il6r*α^*Fl*^ versus *Il6r*α^*KO*^ (*n* = 6–12) mice at day 62 after the 1.5% AOM/DSS protocol, results are presented relative to NCD *Il6r*α^*Fl*^ non-colitic colons. Representative immunofluorescent stainings of **c** FoxP3 (red), **d** γδTCR (red) and **e** IgM (*green*) expressing cells in colons from 17-week-old HFD-fed *Il6r*α^*Fl*^ and *Il6r*α^*KO*^ mice at day 62 of the 1.5% AOM/DSS protocol counterstained with Hoechst 3342. **f** Total number of CD45^+^, F4/80^+^ and F4/80^−^ cells isolated from non-colitic and colitic colons at day 0 or day 13 of the 1.5% AOM/DSS protocol in 8- and 10-week-old NCD-fed *Il6r*α^*Fl*^ (*n* = 4–5) versus *Il6r*α^*KO*^ (*n* = 4) mice. **g** Total number of CD19^+^, γδTCR^+^ and βTCR^+^ cells isolated from non-colitic and colitic colons at day 0 or day 13 of the 1.5% AOM/DSS protocol in 8 and 10-week-old NCD-fed *Il6r*α^*Fl*^ (*n* = 4–5) versus *Il6r*α^*KO*^ (*n* = 4) mice. **h** Total number of CD19^+^CCR-6^+^, γδTCR^+^CCR-6^+^ and βTCR^+^CCR-6^+^ cell isolated from non-colitic and colitic colons at day 0 or day 13 of the 1.5% AOM/DSS protocol in 8- and 10-week-old NCD fed *Il6r*α^*Fl*^ (*n* = 4–5) versus *Il6r*α^*KO*^ (*n* = 4) mice. AOM, azoxymethane; DSS, dextran sodium sulphate; NCD, normal chow diet; HFD, high-fat diet; IPA, Ingenuity pathway analysis; CAC, colitis-associated colorectal cancer. Centre line: median; box limits: 1st and 3rd quartiles; whisker: maximum to minimum, *p ≤ 0.05, **p ≤ 0.01 and ***p ≤ 0.001 two-tailed unpaired Student’s *t*-test **b**, one-way ANOVA followed by Fisher LSD **f**–**h**. Scale bar, 100 μm **c**,** e**, 200 μm **d**

CCR-6, the unique receptor for CCL-20, is mainly expressed on T and B lymphocytes and CCR-6 binding to CCL-20 causes chemoattraction of these cells towards the CCL-20 source^40–43^. In line with this fact, investigation of gene expression data from the microarray demonstrated that lymphocyte-specific genes, such as *Cd19*, *Cd22*, *Cd79A*, *B cell activating factor receptor* (*Baff-R*), *Burton’s tyrosine kinase* (*BTK*) and *paired box protein 5* (*Pax5*) for B cells and *Cd3ε, Cd3δ, Cd4* and *Il2rα* for T cells, were downregulated in IL-6Rα-deficient tumours (Figs. 3a and 4a, Supplementary Table 1). Furthermore, Gene Ontology analysis revealed a reduced quantity of B cells and T cells in CAC in the absence of IL-6Rα signalling (Fig. 4a). To further validate these findings, we performed qPCR and immunohistochemistry of tumours to detect Treg, αβ T cells, γδ T cells and B cells (Fig. 4b–e). Indeed, the qPCR experiments confirmed the reduction of these lymphocyte subsets in IL-6Rα-deficient tumours when compared with controls (Fig. 4b). Notably, these data are in line with our previous observation of reduced lymphocyte counts in IL-6Rα-deficient mice in colitis (Fig. 2h, i). To quantify CCR-6^+^ lymphocyte recruitment in 1.5% AOM/DSS-induced colitis, we examined total cell numbers from non-colitic and colitic control as well as IL-6Rα-deficient colons by FACS (Fig. 4f–h). Although consistent to our previous data, similar macrophage numbers were observed in control and IL-6Rα-deficient colons, IL-6Rα deficiency largely attenuated lymphocyte recruitment in colitis (Fig. 4f). Using specific antibodies in FACS to detect CD19-expressing B cells and γδ as well as αβ T-cell receptor-expressing T cells revealed that these lymphocyte subsets were reduced in the colitis colons of knockout animals (Fig. 4g). Furthermore, co-staining with CCR-6 antibody revealed that the majority of B cells and γδ T cells in colitis express CCR-6, whereas only a minority of αβ T cells express the CCR-6 receptor (Fig. 4h) when compared with their total numbers (Fig. 4g). Taken together, these findings clearly demonstrate the reduced presence of CCR-6-expressing lymphocytes in IL-6Rα-deficient CAC and imply an impaired chemoattractance of these cells into the CAC TME of IL-6Rα-deficient animals.

### CCR-6^+^ lymphocytes promote CAC development

**Fig. 5:**
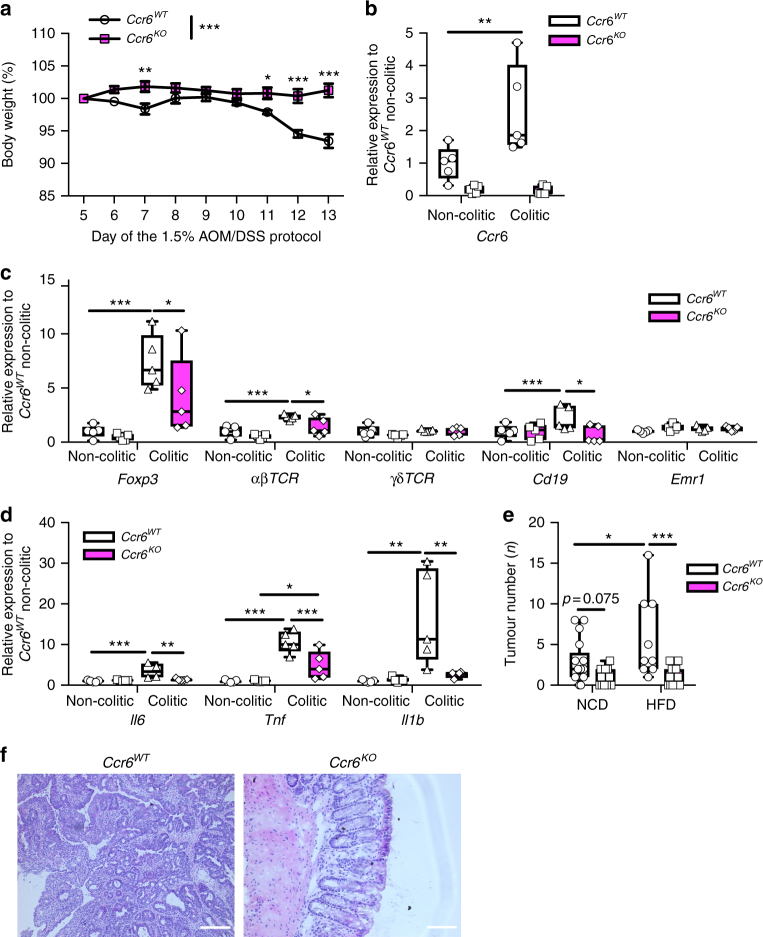
CCR-6^+^ lymphocytes promote CAC. **a** Body weight loss in % of NCD-fed *Ccr6*^*WT*^ (*n* = 10) and *Ccr6*^*KO*^ (*n* = 11) mice at day 5 to day 13 of the 1.5% AOM/DSS protocol. **b**–**d** qPCR analysis of indicated gene expression in non-colitic and colitic colons of 8- and 10-week-old NCD-fed control *Ccr6*^*WT*^ and *Ccr6*^*KO*^ at day 0 and day 13 of the 1.5% AOM/DSS protocol (*n* = 5), results are presented relative to non-colitic *Ccr6*^*WT*^ colons. **e** Tumour number of 17-week-old NCD (*n* = 17–13) and HFD (*n* = 9–12)-fed control *Ccr6*^*WT*^ and *Ccr6*^*KO*^ mice counted at day 62 of the 1.5% AOM/DSS protocol. **f** H&E staining of colons from NCD-fed 17-week-old *Ccr6*^*WT*^ and *Ccr6*^*KO*^ mice at day 62 of the 1.5% AOM/DSS protocol. AOM, azoxymethane; DSS, dextran sodium sulphate; NCD, normal chow diet; HFD, high-fat diet; CAC, colitis-associated colorectal cancer. Data are represented as mean + SEM or centre line: median; box limits: 1st and 3rd quartiles; whisker: maximum to minimum, *p ≤ 0.05, **p ≤ 0.01 and ***p ≤ 0.001 two-way ANOVA followed by Fisher LSD **a**–**e**. Scale bar, 50 μm

To directly examine whether the CCL-20/CCR-6 axis is necessary for lymphocyte recruitment during colitis, we investigated CCR-6-deficient mice (*Ccr6*^*KO*^) in our CAC model^44^. During the colitis phase, CCR-6-deficient mice exhibited reduced weight loss (Fig. 5a). Importantly, colons from colitic control *Ccr6*^*WT*^ mice had increased *Ccr6* expression at day 13 compared with non-colitic controls, whereas consistently *Ccr6* expression was absent in *Ccr6*^*KO*^ mice (Fig. 5b). Colitic colons derived from *Ccr6*^*KO*^ animals had reduced expression of markers for Tregs, αβ T cells, and B cells, but not of γδ T cells and macrophages (Fig. 5c). Concomitantly, expression of *Il6*, *Tnf* and *Il1β* were reduced in colitis colons of CCR-6-deficient mice (Fig. 5d). Thus, these analyses demonstrate that CCR-6-expressing cells are recruited during colitis and that CCR-6 deficiency largely protects against colitis. Consistently, CCR-6-deficient animals failed to develop CAC, both under lean and obese conditions (Fig. 5e, f, Supplementary Fig. 5a-c). Therefore, we conclude that CCL-20 released in the colitis phase recruits CCR-6-expressing lymphocytes that promote CAC tumourigenesis. However, which CCR-6-expressing lymphocyte population, that is recruited to the colon in colitis promotes CAC, still has to be investigated. B cells, γδ T cells and Tregs are known to express CCR-6^40-43^.

### Recruitment of B cells via CCL-20/CCR-6 promotes CAC

**Fig. 6:**
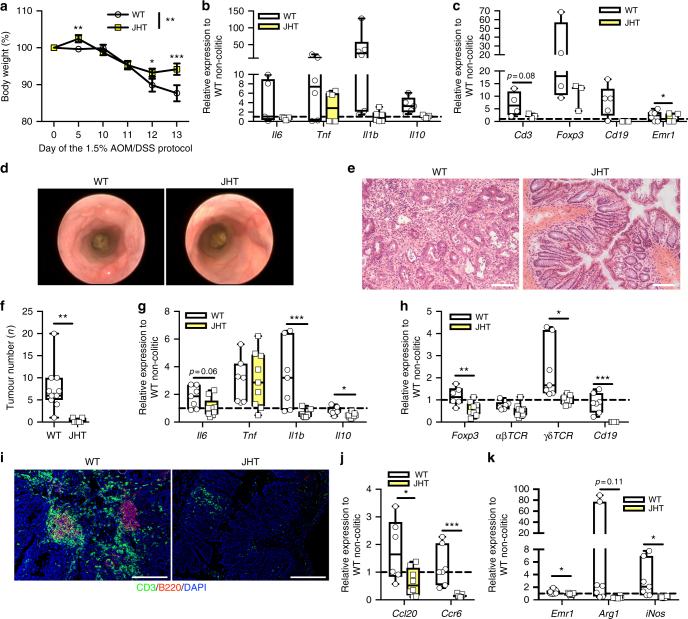
Recruitment of CCR-6-expressing B cells alters the TME to promote CAC. **a** Body weight loss in % of NCD-fed WT (*n* = 17) and JHT (*n* = 14) mice at day 0 to day 13 of the 1.5% AOM/DSS protocol. **b**, **c** qPCR analysis of indicated gene expression in colitic colons of 10-week-old NCD-fed WT and JHT (*n* = 3–9) mice at day 13 of the 1.5% AOM/DSS protocol, results are presented relative to non-colitic WT colons at day 0. **d** Representative endoscopic pictures of 13-week-old NCD-fed WT and JHT mice of the 1.5% AOM/DSS protocol. **e** H&E staining of colons from NCD-fed 17-week-old WT and JHT mice at day 62 of the 1.5% AOM/DSS protocol. **f** Tumour number of 17-week-old NCD-fed WT (*n* = 11) and JHT (*n* = 7) mice counted at day 62 of the 1.5% AOM/DSS protocol. **g**, **h** qPCR analysis of indicated gene expression in colons of 17-week-old NCD-fed WT (*n* = 7) and JHT (*n* = 9) mice at day 62 of the 1.5% AOM/DSS protocol, results are presented relative to non-colitic WT colons at day 0. **i** Representative immunofluorescent stainings of CD3 (green)-expressing T cells and B220 (red)-expressing B cell in colons of 17-week-old NCD-fed WT and JHT mice at day 62 of the 1.5% AOM/DSS protocol. **j**, **k** qPCR analysis of indicated gene expression in colons of 17-week-old NCD-fed WT (*n* = 7) and JHT (*n* = 9) mice at day 62 of the 1.5% AOM/DSS protocol, results are presented relative to non-colitic WT colons at day 0. AOM, azoxymethane; DSS, dextran sodium sulphate; NCD, normal chow diet; CAC, colitis-associated colorectal cancer; TME, tumour microenviroment; JHT, targeted deletion of the JH locus. Data are represented as mean + SEM or centre line: median; box limits: 1st and 3rd quartiles; whisker: maximum to minimum, *p ≤ 0.05, **p ≤ 0.01 and ***p ≤ 0.001 two-tailed unpaired Student’s *t*-test **f** or two-way ANOVA followed by Fisher LSD **a**,** b**,** c**,** g**,** h**,** j**,** k**. Scale bar, 50 μm **e**, 200 μm **i**

Next, we aimed at investigating which CCR-6^+^ cell type contributes to CAC development. A prominent CCR-6-expressing lymphocyte population comprises mature B cells^41,45^ and B cells exert macrophage-polarising functions in colitis. In particular, regulatory B1b-like B cells in the colon (CD5^−^, IgM^+^, CD19high, B220low) provide IL-10 to polarise macrophages towards M2-like phenotype^46,47^ and such B cells are induced by gut microbiota-driven IL-1β and IL-6 production^48^. Presumably, these B cells sense and control commensal bacteria during DSS-induced colonic damage^49^ and engaging innate toll like receptor (TLR)4 on B cells by LPS causes IL-6 production^50^. Thus, B cells can exert numerous activities on the CAC TME that are not directly committed to humoral immunity. To investigate the role of B cells and their recruitment via CCR-6/CCL-20 in CAC, we employed B cell-deficient mice. B cells rearrange their VDJ genes during B cell development and removal of J elements via gene targeting in mice results in B cell-deficient mice (JHT mice)^51^. We subjected B cell-deficient JHT mice to our CAC protocol and examined colitis parameters and CAC tumour development. Body weight loss in colitis and colitis-induced expression of *Il6*, *Tnf*, *Il1β* and *Il10* were reduced in JHT mice (Fig. 6a, b). Furthermore, besides the lack of B cells, less immune cells such as T cells, Tregs and macrophages were present in colitis colons of JHT mice (Fig. 6c). This is in line with the finding that control mice developed dysplastic lesions, whereas the absence of B cells ameliorated colon pathology as revealed by endoscopy (Fig. 6d). Ultimately, control mice developed CAC, whereas B cell deficiency largely protected against CAC (Fig. 6e, f). Investigation of gene expression revealed that JHT mice exhibited decreased colonic expression of *Il6*, *Il1β* and *Il10,* whereas *Tnf* was unaltered (Fig. 6g). Furthermore, not only B cells were absent in JHT colons but also gene expression markers for T cells and Tregs were reduced (Fig. 6h, i). Consistent with this fact was a reduced *Ccr6* expression accompanied with a decreased *Ccl20* expression in JHT colons, suggesting that B cells ultimately impact on the CAC TME (Fig. 6j). Apparently, the decreased *Ccl20* expression is in line with an impaired macrophage polarisation to *Arg1*-expressing M2 as well as *iNos*-expressing M1-like in JHT colons (Fig. 6k). Collectively, these analyses reveal that during colitis CCL-20-recruited CCR-6^+^ B cells promote CAC. Contrariwise, we have also investigated JHT mice in the CAC protocol under obese conditions where B cell-deficient mice died either spontaneously or had to be killed owing to excessive colitis symptoms (Supplementary Fig. 5d, e). This discrepancy between diets in B cell-deficient mice hints to a more complex and dual role for B cells in colonic inflammation under different dietary exposures than hitherto assumed and even more strengthens our finding that CCR-6-expressing B cells affect CAC.

### γδ T-cell inhibition ameliorates CAC

**Fig. 7:**
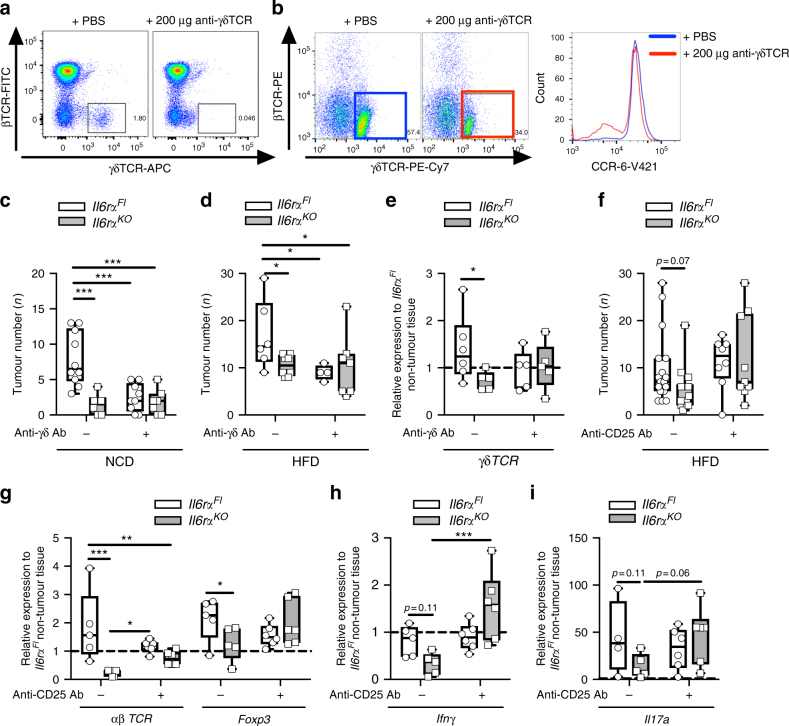
Recruitment of CCR-6^+^ T cells in CAC. **a** Representative FACS plots of βTCR and γδTCR expression in blood of NCD-fed *Il6r*α^*Fl*^ mice at day 5 injected either with PBS or 200 μg anti-γδ antibody at day 3 of the 1.5% AOM/DSS protocol. **b** Representative FACS plots of βTCR and γδTCR expression in lymphocytes of colons derived from 10-week-old NCD fed *Il6r*α^*Fl*^ mice at day 13 injected either with PBS or 200 μg anti-γδ antibody at day 3 of the 1.5% AOM/DSS protocol and histogram of mean fluorescent intensity of CCR-6 expression in γδTCR^+^ lymphocytes from PBS (blue) or 200 μg anti-γδ antibody (red) injected mice. Tumour number of 17-week-old NCD **c** (*n* = 6–11) and HFD-fed **d** (*n* = 4–8) of *Il6r*α^*Fl*^ and *Il6r*α^*KO*^ mice counted at day 62 injected with either PBS or 200 μg anti-γδ antibody at day 3 of the 1.5% AOM/DSS protocol. **e** qPCR analysis of *γδTCR* gene expression in tumours of 17-week-old NCD-fed *Il6r*α^*Fl*^ and *Il6r*α^*KO*^ mice (*n* = 6) at day 62 injected with either PBS or 200 μg anti-γδ antibody at day 3 of the 1.5% AOM/DSS protocol, results are presented relative to *Il6r*α^*Fl*^ non-tumour tissue. **f** Tumour number of 17-week-old HFD-fed *Il6r*α^*Fl*^ and *Il6r*α^*KO*^ mice (*n* = 8–17) counted at 62 days injected with either PBS or 1 mg anti-CD25 antibody at day 3 of the 1.5% AOM/DSS protocol. **g**–**i** qPCR analysis of indicated gene expression in tumours of 17-week-old HFD-fed *Il6r*α^*Fl*^ and *Il6r*α^*KO*^ mice (*n* = 6–7) at day 62 injected with either PBS or 1 mg anti-CD25 antibody at day 3 of the 1.5% AOM/DSS protocol, results are presented relative to *Il6r*^*Fl*^ non-tumour tissue. AOM, azoxymethane; DSS, dextran sodium sulphate; NCD, normal chow diet; HFD, high-fat diet; CAC, colitis-associated colorectal cancer; Ab, Antibody. centre line: median; box limits: 1st and 3rd quartiles; whisker: maximum to minimum, *p ≤ 0.05, **p ≤ 0.01 and ***p ≤ 0.001 two-way ANOVA followed by Fisher LSD **c**–**i**

Although we have demonstrated a crucial function of CCR-6-expressing B cells in the CAC TME, also T cells such as γδ T cells express the CCR-6 receptor. γδ T cells can be separated into CCR-6- and NK1.1-expressing subtypes that fulfil T helper (Th) 17 and Th1 cell functions, respectively^43^. Koenecke et al. demonstrated that anti-γδ T-cell receptor (TCR) antibody injection in mice yielded inhibition of γδ T cells because they downregulate their γδTCR^52^. We aimed at addressing the function of γδ T cells and their chemoattraction via CCL-20 in our CAC model by injecting anti-γδTCR at day 3 of the 1.5% AOM/DSS protocol prior to the colitis phase of CAC into control and IL-6Rα-deficient animals. Anti-γδTCR injection led to downregulation of γδTCR as obvious from the appearance of CD3^+^ αβ T cells and γδ T cells in the blood of these mice (Fig. 7a). Although most γδ T cells express CCR-6 during colitis in untreated mice, a proportion of γδ T cells in antibody-treated mice were negative for CCR-6 expression (Fig. 7b). Ultimately, lean control mice developed CAC, whereas γδTCR antibody injection drastically reduced tumour burden in control mice similar to IL-6Rα-deficient mice (Fig. 7c, Supplementary Fig. 6a-c). Notably, the reduced CAC burden in anti-γδTCR-treated control mice was largely recapitulated under obese conditions (Fig. 7d, Supplementary Fig. 6d-f). Analysis of tumour-derived gene expression revealed slightly reduced *γδTCR* upon anti-γδTCR antibody injection in lean mice (Fig. 7e). Our experiments are in line with previous reports assigning γδ T cells as crucial mediators in colonic inflammation by providing pathogenic IL-17^53^. Thus, recruitment of CCR-6^+^ γδ T cells promotes CAC and inhibition of γδ T cells might be a reasonable therapeutically strategy against CAC.

### Treg depletion restores CAC in obese IL-6Rα-deficient mice

Another CCR-6-expressing T lymphocyte population that has been demonstrated to contribute to CAC are Treg cells, which control gut inflammation via their immunosuppressive functions^37^. We have shown that IL-6 signalling in effector T cells is required to release them from Treg-mediated suppression using the well-established Ovalbumin (OVA) model^34^. Suppression of effector function in IL-6Rα-deficient cells included a blunted Th1/IFNγ and total absence of Th17 IL-17a response. However, the Th1 response could be restored in IL-6Rα-deficient T cells when Tregs were depleted by anti-CD25 antibody treatment. In order to examine whether Tregs also suppress effector functions in lean and obese IL-6Rα-deficient mice in our CAC model, anti-CD25 antibody was i.p. injected at day 3 of the 1.5% AOM/DSS protocol just prior to the colitis phase of CAC. This experimental procedure should provide only a transient Treg depletion during the colitis phase and should affect T effector cells to a lesser extent that upon activation upregulate CD25. Most likely, the single anti-CD25 antibody injection depletes Tregs only until anti-CD25 antibody is consumed/degraded where in the context of proceeding T-cell development these cells are able to repopulate the colon. Nevertheless, although in lean mice, anti-CD25 antibody-mediated transient depletion of Treg cells exhibited only minor effects, it restored CAC development in obese IL-6Rα-deficient animals, whereas obese control mice had unaltered CAC (Fig. 7f, Supplementary Fig. 6g-m). Detection of markers for αβ T cells and Treg cells in tumours of the obese cohorts revealed a reduction of these cells in IL-6Rα-deficient mice that was largely unaltered when mice received anti-CD25 treatment (Fig. 7g). However, despite the fact that also other cells express *Ifnγ* and *Il17a*, the examination of T-cell effector molecules revealed decreased *Ifnγ* and *Il17a* expression in obese IL-6Rα-deficient tumours that have been restored upon Treg depletion (Fig. 7h, i). Thus, Treg depletion in obese IL-6Rα-deficient mice restores CAC development via restoration of T-cell effector functions.

**Fig. 8:**
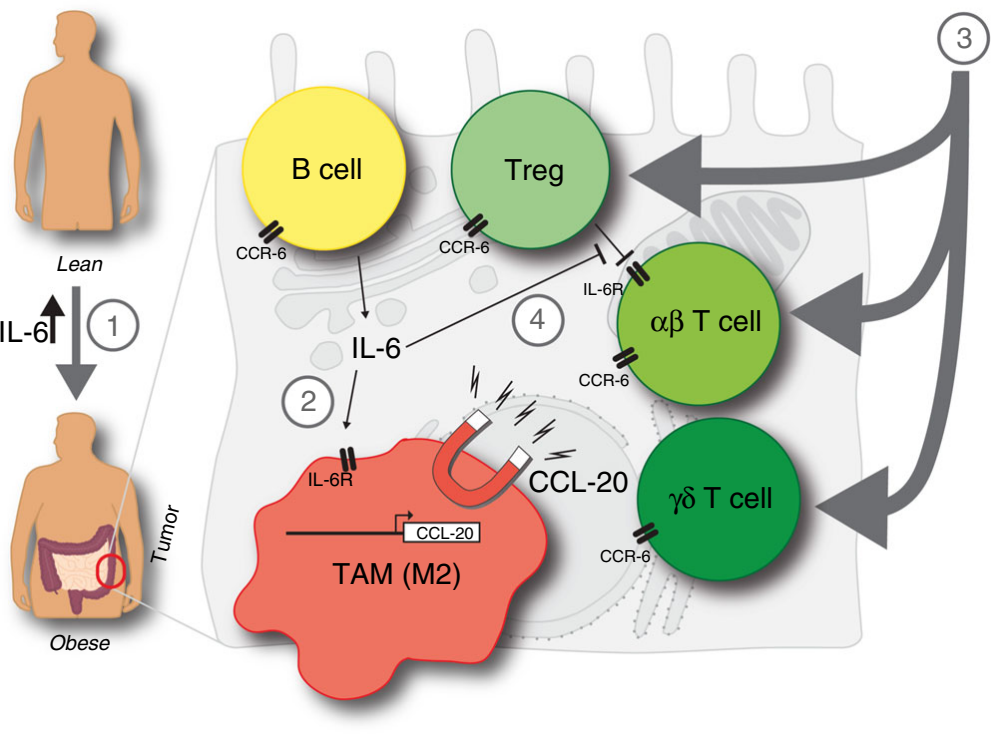
Mechanism how obesity and IL-6 alter the colitis-associated tumour microenvironment. 1. Obesity-induced gut barrier defects cause a low-grade inflammation including increases in IL-6 levels. 2. IL-6 polarises macrophages towards M2-type in colitis-associated colorectal cancer (CAC) that in response to commensal factors express the chemoattractant CCL-20. 3. CCL-20 recruits CCR-6-expressing lymphocytes comprising B cells, regulatory T cells (Treg) and T cells expressing αβ and γδ T-cell receptors that impact on CAC progression. B cells impact on macrophage polarisation, whereas CCR-6^+^ γδ T cells provide pathogenic IL-17. 4. IL-6 signalling in αβ T cells releases them from Treg-mediated suppression to exert their effector functions such as T helper (Th)1 and Th17 responses. TAM, tumour-associated macrophage

Collectively, these experiments reveal a crucial role for the recruitment of CCR-6-expressing lymphocytes into the colon during the colitis phase to promote CAC. Knockout and antibody-inhibition/depletion models demonstrate that recruitment of CCR-6-expressing B cells and γδ T cells promote CAC development whereas CCR-6^+^ Tregs exert an immunosuppressive role in CAC under obese conditions by interfering with T-cell effector functions. B cells and IL-6 synergise in the polarisation towards CCL-20-expressing macrophages causing a vicious circle of CCR-6^+^ lymphocyte recruitment in CAC (Fig. 8). Overall, our experiments assign obesity-induced IL-6 an unappreciated role in the CAC TME by regulating macrophage polarisation and lymphocyte recruitment.

## Discussion

The steadily increasing obesity epidemic and the simultaneous incidence of obesity-associated comorbidities such as colorectal cancer requires a detailed understanding of molecular and cellular mechanisms that are affected to develop potential novel therapies. Here, we show that obesity exacerbates AOM/DSS-induced CAC via increased expression of inflammatory cytokines. However, our experimental set up was unable to address whether in obesity pre-neoplastic lesions occur earlier than in lean mice. We demonstrate that obesity-induced impairment of gut barrier function recruits and activates immune cells that promote CAC. In the colitis phase of CAC, IL-6-polarised M2-like macrophages express the chemokine CCL-20 that recruits CCR-6-expressing lymphocytes, further promoting CAC progression. Our in vitro experiments suggest that intestinal M2 macrophages express CCL-20 in colitis via exposure to commensal antigens such as LPS and not as a consequence of direct IL-6-activated transcriptional traits. Though IL-6-type cytokine-activated STAT3 can directly activate CCL-20 expression^54^, CCL-20 expression is mainly regulated via inflammatory signalling and nuclear factor kappa-light-chain-enhancer of activated B cells (NF-κB)-controlled transcription^55–58^. Given that LPS-activated NF-κB-controlled gene expression in M1 macrophages has only minor effects on CCL-20 expression suggests extra transcription factors active in M2 macrophages that act in concert with LPS-induced NF-κB to increase CCL-20 expression. Hence, the substantially reduced *Ccl20* expression in colons of *Il6rα*^*KO*^ mice is a consequence of collapsed M2-type polarisation of IL-6Rα-deficient macrophages in CAC, thereby compromising CCR-6-expressing lymphocyte recruitment in colitis to promote tumourigenesis. CCR-6-expressing B cells, γδ T cells and Treg cells^40,43^ have either low or undetectable IL-6Rα expression underlining our observation that the protection against CAC in IL-6Rα-deficient mice is not a direct consequence of IL-6 action in these cells but is rather an indirect effect through compromised chemoattraction. Nevertheless, these cells might still be able to receive IL-6 signalling in control mice via IL-6-transsignalling mechanisms that we have avoided by using IL-6Rα-deficient mice devoid of both membrane and soluble signalling capacities. Despite the crucial role of direct IL-6 function on the approval of Treg-mediated suppression of αβ T cells, we estimate that Tregs also inhibit other immune cells by a similar mechanism as T-cell-specific inactivation of IL-6 signalling using CD4-Cre is unable to prevent CAC development. In line with this assumption, γδ T cells in the colon were known to be suppressed via Tregs and γδ T cells are not affected by CD4-Cre-mediated recombination^59^. Although γδ T cells can promote CRC via allocation of IL-17 to transformed tumour cells^60,61^, they are also able to restrain αβ T-cell responses in cancer by providing inhibitory programmed cell death 1 ligand to αβ T cells^62^.

Collectively, our study assigns obesity-induced IL-6 as a modulator of the TME in CAC via macrophage polarisation and successive lymphocyte recruitment via the CCL-20/CCR-6 axis. In light of the steadily increasing obesity epidemic, novel treatment options to combat obesity-associated disorders are desperately required highlighting our study to interfere with inflammatory signalling and to inhibit corrupt cell types as important contribution to global health issues.

## Methods

### Animals

All animal procedures were in compliance with protocols approved by local government authorities (Bezirksregierung, Cologne, Germany Az: 8.87-50.10.31.08.279, 84-02.04.2014.A211 and Mainz, Germany G16-1-072) and were in accordance with NIH guidelines. The experimental cohorts of mice (mus musculus) were females (if not stated differently) that were housed in four different specific pathogen free animal facilities under similar conditions. Mice were housed in single ventilated cages (IVCs, TypII long) in groups of three to five at 22–24 °C in a 12-hour–12-hour light-dark cycle (with lights on at 7:00 AM). The cages were implemented with bedding material (Lignocel FS14) and cotton nestlet material (Plexx, 14010). Mice were fed a NCD (Teklad Global Rodent T.2018.R12; Harlan) containing 53.5% carbohydrates, 18.5% protein and 5.5% fat (12% of calories from fat) or, from 3 weeks of age, were fed a HFD (C1057; Altromin) containing 32.7% carbohydrates, 20% protein and 35.5% fat (55.2% of calories from fat). Food and water was available ad. libitum. The conditional *II6rα* mouse strain served as control mice (*II6rα*^*Fl*^)^20^. Mice bearing a complete (*II6rα*^*KO*^)^22^, myloid-specific (*Il6rα*^*myl-KO*^)^19^, intestinal epithelial-specific (*Il6rα*^*IEC-KO*^)^27^ and T cell-specific (*Il6rα*^*T-KO*^)^34^ knockout were generated and genotyped. For identifying the *II6rα*^*Fl*^ and the *II6rα*^*KO*^ alleles PCR was performed with the following primers: 5GK12 (5′-CCGCGGGCGATCGCCTAGG-3′), 5IL6Rex3 (5′-CCAGAGGAGCCCAAGCTCTC-3′) and 3IL6RA (5′-TAGGGCCCAGTTCCTTTAT-3′). The following primers were used to genotype the transgenes LysM-Cre LysMCre1 (5′-CTCTAGTCAGCCAGCAGCTG-3′) and LysMCre2 (5′-ATGTTTAGCTGGCCCAAATGT-3′), Villin-Cre VilCre1 (5′-ACAGGCACTAAGGGAGCCAATG-3′), VilCre2 (5′-ATTGCAGGTCAGAAAGAGGTCACAG-3′) and VilCre3 (5′-GTTCTTGCGAACCTCATCACTC-3′) and CD4-Cre CD4Cre1 (5′-CCCAACCAACAAGAGCTC-3′) and CD4Cre2 (5′-CCCAGAAATGCCAGATTACG-3′). C57BL/6 N (#027) and C57BL/6 J (#N/A) mice were obtained from Charles River, JHT^51^ and *Ccr6*^KO^ (#013061)^44^ mice from Jackson laboratories. All animals were on a C57BL/6 N genetic background, except *Ccr6*^KO^ and their control *Ccr6*^*WT*^, they were on C57BL/6 J genetic background.

### Induction of CAC in AOM/DSS model and cell depletion

 2.5% AOM/DSS protocol: 8-week-old mice fed a NCD or a HFD were i.p. injected with 10 mg/kg body weight AOM (A5486, Sigma-Aldrich) at day 1 of the 2.5% AOM/DSS protocol (Supplementary Fig. 1f). The animals were exposed to three repetitive cycles of 2.5% DSS (MW = 36,000–50,000, 0216011080, MP Biomedicals) in the drinking water for 7 days from day 1–7, day 22–28 and day 43–49 (DSS water was refreshed every second day) to induce colitis. Animals were killed by CO_2_ with 17 weeks of age at day 62 for tumour analysis. Mice that lost more than 20% of their body weight during the colitic phases were killed.

 1.5% AOM/DSS protocol: 8-week-old NCD or HFD-fed mice were i.p. injected with 10 mg/kg body weight AOM (A5486, Sigma-Aldrich) at day 1 of the 1.5% AOM/DSS protocol (Fig. 1c). At day 5 mice were exposed to 1.5% DSS (MW = 36,000–50,000, 0216011080, MP Biomedicals) in the drinking water for 5 days (DSS water was refreshed at day 7) and provided with normal drinking water from day 10 on until the end of the experiment at day 62. Animals were killed with 8 weeks at day 0 for non-colitic control tissue or at day 13 for colitic analysis or at day 62 for tumour analysis. The colon length was measured from caecum till the anus. The distal part of the colon was used for non-colitic and colitic analysis. At day 62 tumours were counted in a blinded fashion by number, size (<2 mm, >2 mm) and percentage of the afflicted area of the distal colon. Tumour and non-tumour tissue from the distal colon was separated and used for tumour analysis.

To neutralise IL-6 and sIL-6R signalling, 8-week-old C57BL/6 animals were injected i.p. with 500 µg anti-IL-6 antibody (BE0046. BioXCell) or 150 µg sGP130Fc (provided by Christoph Garbers, Kiel University, Germany) at day 3 of the 1.5% AOM/DSS protocol, respectively.

To deplete γδTCR-expressing and Treg cells, *II6rα*^*Fl*^ and *II6rα*^*KO*^ mice were injected with 200 µg anti-γδ antibody (UC7-13D5, eBioscience) or 1 mg anti-CD25 antibody (BE0013, BioXCell) at day 3 of the AOM/DSS protocol, respectively. Control animals were injected with the same volume of PBS.

### Endoscopy

Non-invasive endoscopy (TRICAM endoscope Karl Storz) was performed under either i.p. injection of ketamine/Rompun or inhalation narcosis using isoflurane with 13-week-old animals.

### Analysis of body composition

Fat mass was determined via nuclear magnetic resonance (NMR Analyser minispeq mq7.5; Bruker Optik, Ettlingen, Germany) in 17-week-old mice.

### Glucose-tolerance test

Glucose-tolerance tests were done with 7–8-week-old C57BL/6 mice. Mice were fasted overnight for 16 h and glucose concentrations in blood were measured after the fasting period. Then each animal received an i.p. injection of 20% glucose solution (10 ml/kg body weight) and glucose concentrations in blood were measured after 15, 30, 60 and 120 min. Glucose concentrations in whole venous blood were measured with an automatic glucose monitor (Bayer Contour; Bayer).

### Analytical procedures

The concentrations of leptin and insulin in serum, CCL-20 in the supernatent of cultured BMDM and albumin in the faeces were measured by enzyme-linked immunosorbent assays, with mouse standards, according to manufacturer’s guidelines.: Mouse Leptin ELISA (90030, Crystal Chem), Mouse Insulin ELISA (90080, Crystal Chem), Mouse CCL-20/MIP-3 alpha Quantikine ELISA Kit (MCC200, R&D Systems) and Mouse Albumin ELISA Quantification set (E90-134,Bethyl).

### Generation of BMDMs

NCD-fed mice were killed by cervical dislocation and bone marrow was isolated from femurs and tibias. Bone marrow cells were plated in RPMI-1640 medium (supplemented with 10% fecal calf serum (FCS), 1% glutamine, 1% penicillin-streptomycin and 10–50 ng/ml macrophage colony-stimulating factor (130-101-706, Miltenyi) and were allowed to differentiate for 7 days. At 24 h before all experiments, macrophage colony-stimulating factor was removed and cells were washed two times with sterile PBS. BMDMs were either stimulated 24 h with 20 ng/ml IFNγ (130-094-048, Miltenyi) and 100 ng/ml LPS (*Escherichia coli* strain O55:B5; Sigma) or 20 ng/ml IL-4 (130-097-757, Miltenyi) and 50 ng/ml IL-6 (130-096-685, Miltenyi) to obtain M1- and M2-polarised macrophages, respectively. Polarised M1 and M2 were washed with PBS and then stimulated for 8 h or 24 h with 50 ng/ml IL-6 and 10 ng/ml LPS.

### MACS sorting

Immune cells and IECs from the colon were isolated according to recent protocol^63^. IECs were removed from the upper phase after the percoll gradient, washed twice with PBS containing 2% FCS. To purify macrophages, the percoll separated immune cells were used for magnetic separation with the anti-F4/80 MicroBeats UltraPure (130-110-443, Miltenyi) following the instruction manual. The IEC and macrophages were shock frozen in liquid nitrogen and kept at −80 degrees until further use.

### Flow cytometry

Immune cells from the colon were isolated according to recent protocol^63^. The isolated immune cells were resuspended in FACS buffer (2% FCS in PBS) and were passed through a 70-μm strainer (BD Biosciences). Samples were analysed with following antibodies: anti-CD11b-PE (1:100 dilution, 101208, Biolegend), anti-CD11c-PE-Cy7 (1:100 dilution, 117318, Biolegend), anti-γδTCR-APC (1:100 dilution, 118116, Biolegend), anti-γδTCR-PE-Cy7 (1:100 dilution, 118123, Biolegend), anti-βTCR-FITC (1:100 dilution, 109205, Biolegend), anti-βTCR-PE (1:100 dilution, 553172, BD Biosciences), anti-CD45-V510 (1:100 dilution, 103137, Biolegend), anti-CD45-PE-CF594 (1:200 dilution, 562420, BD Biosciences), anti-CD45-APC (1:200 dilution, 103112, Biolegend), anti-CD90.2-PE-Cy7 (1:200 dilution, 140309, Biolegend), anti-CD3-PE-CF594 (1:200 dilution, 562332, BD Biosciences), anti-F4/80-APC-Cy7 (1:50 dilution, 123119, Biolegend), anti-CD19-FITC (1:100 dilution, 115505, Biolegend), anti-CCR-6-V421 (1:50 dilution, 129817, Biolegend), anti-IL-6R-PE (1:50 dilution, 115805, Biolegend) and LIVE/DEAD Fixable Violet Dead Cell Stain Kit or LIVE/DEAD Fixable Aqua Dead Cell Stain Kit (1:1000 dilution, L34955 and L34957, ThermoFisherScientific). Data were acquired on MACSQuant Analyzer (Miltenyi) and MACSQuant VYB (Miltenyi) and data were analysed with FlowJo software (Treestar).

### Immunoblot analysis

Tissues were homogenised in protein lyses buffer with FastPrep-24 (MP Biomedicals) and centrifuged at 13,000 g for 1 h at 4°C. Proteins were separated by SDS-polyacrylamide gel electrophoresis (10%) and transferred to PVDF membranes (Bio-Rad). Membranes were probed with the following antibodies: Calnexin (1:5000 dilution, 208880, Calbiochem), PCNA (1:1000 dilution, D3H8P, 13110, CellSignalling).

### Analysis of gene expression

Total RNA isolated from colons, tumour tissue, T cells, BMDMs and primary macrophages was analysed by qPCR. RNA was isolated from tissue and cells with an RNeasy Kit (74106, Qiagen) or Arcturus PicoPure (KIT0214, Applied Biosystems) and treated with DNase. The RNA was reverse-transcribed with a High Capacity cDNA RT Kit (4368813, Applied Biosystems) and was amplified with TaqMan Gene Expression Master Mix (4369542, Applied Biosystems). The following primers from ThermoFisherscientific were used: *Arg1* (Mm00475988_m1), *Ccl5* (Mm01302428_m1), *Ccl20* (Mm01268754_m1), *Ccr1* (Mm00438260_s1), *Ccr3* (Mm00515543_s1), *Ccr6* (Mm99999114_s1), *Cd3d* (Mm00442746_m1), *Cd19* (Mm00515420_m1), *Emr1* (Mm00802529_m1), *Foxp3* (Mm00475165_m1), *Il1b* (Mm01336189_m1), *Il6* (Mm00446190_m1), *Il10* (Mm00439616_m1), *Il17* (Mm00439619_m1), *Ill4r* (Mm01275139_m1), *Il6ra* (Mm00439649_m1), *Ifng* (Mm01168134_m1), *Nos2* (Mm00440485_m1), *Tnf* (Mm00443260_g1), Tata box binding protein (*Tbp*) (Mm00446971_m1). *Tbp* was used as reference gene. Self-designed exon spanning probes for the constant region of the αTCR chain and the highly homologous 1, 2 and 3 γTCR were obtained from Eurogentec including forward and reverse primer as well as 5’-FAM/3’-TAMRA labelled probes: αTCR-fwd 5′-AAAACTGTGCTGGACATGAA-3′, αTCR-rev 3′-CATCACAGGGAACGTCTGAA-5′, and αTCR-probe 5′-FAMAGATATCTTGGCAGGTGAAGCTT-3′ as well as γTCR-fwd 5′-CTGGCAAGATAAAAATGATGTG-3′, γTCR-rev 3′-ACAGATGTTCTTCTAAGCAGA-5′, and γTCR-probe 5′-ACCTCTGCCTACTACACCTACCT-3′. The expression of each specific mRNA analysed was adjusted for total RNA content by comparison with qPCR analysis of mRNA encoding *Tbp*. Results were calculated by the ‘change-in-cycling-threshold’ (ΔCt) comparative method (as 2^−ΔΔCt^). An ABI-PRISM 7900 HT Sequence Detection system (Applied Biosystems) was used for qPCR.

### Microarray

For microarray expression data, RNA was isolated from tumour tissue of colons of *Il6rα*^*Fl*^ and *Il6rα*^*KO*^ HFD-fed mice and further processed to be hybridised to GeneChip Mouse Gene 1.0 ST Arrays (Affymetrix) according to manufacturer's instructions^19^. Affymetrix Powertools and the robust multiarray average method were used for background correction, quantile normalisation and summarisation of the raw intensity values. R software (version 2.13.1) along with the Bioconductor software package were used for the robust multiarray average method, statistical analysis (Student’s *t*-test), calculation of the change in intensity and illustrations. Genes were considered significantly deregulated when they had a difference in expression with a *P* value of ≤ 0.025. Genes with a fold change of ≤ 1.5 were removed from the data.

### Immunohistochemistry

Colons were fixed in 4% formalin and embedded in paraffin. Paraffin sections of the colon were deparaffinised. Macrophages and T cells in the colon were stained with primary antibodies anti-F4/80 (1:500 dilution, MCA 4976, Serotec) or anti-CD3 (1:100 dilution, 5690, Abcam) and with secondary antibodies anti-rat-Biotin-SP (1:500 dilution, 112-065-003, Jackson) or AP-conjugated goat anti-rabbit (1:500 dilution, A3687, Sigma), respectively. Stainings were visualised with either ABC Kit Vectastain Elite (Vector Laboratories) and DAB substrate (Vector Laboratories) or Vector blue (Vector Laboratories). Colons were counterstained with haematoxylin or eosin according to standard protocol and imaged with AxioVision 4.2 (Carl Zeiss MicroImaging). Proliferating IEC were stained with anti-Ki67 (1:200 dilution, ab15580, Abcam), counterstained with 4',6-diamidino-2-phenylindole (DAPI) in the mounting medium (Vectashield) and imaged with Axio Imager 2 (Carl Zeiss MicroImaging). Immunoreactive density of Ki67 and DAPI-positive cells was set in relation to immunoreactive density of all DAPI-positive cells using Fuji.

Fluorescent immunohistochemistry of 4% paraformaldehyde-fixed cryosections was performed using the tyramide signal amplification (TSA) Fluorescence/ Cyanine 3 system (Fluorescein NEL701 A, Cyanine NEL 704 A, PerkinElmer) and a fluorescence microscope (IX70; Olympus or Leica SP8, Leica) using primary antibodies against F4/80 (1:100 dilution, AP10243P4-5, Acris), IgM biotin (1:100 dilution, 98673, Abcam), FoxP3 (1:100 dilution, 14-5773-82, eBioscience), CD3 (1:100 dilution, 16-0031-82, eBioscience), B220 (1:100 dilution, 553092, BD Bioscience), CCL-20-Allophycocyanin conjugated (1:50 dilution, IC760A, R + D Systems) and γδTCR 1:100 dilution, 13-58811-81, eBioscience) respectively. Nuclei were counterstained with Hoechst 3342 (Invitrogen) or mounting medium for fluorescence with 4,6-diamidino-2-phenylindole (H-1200, Vector). In brief, cryosections were fixed in 4% PFA for 20 min followed by sequential incubation with methanol, avidin/biotin (Vector Laboratories) and protein blocking reagent (T144.1 Roti-ImmunoBlock, Roth) to eliminate unspecific background staining. Slides were then incubated overnight with primary antibody specific for the respective antigen. Subsequently, the slides were incubated for 30 min at room temperature with biotinylated secondary antibodies (1:300 dilution, 127-065-160 Jackson Immunoresearch and 554014, BD Pharmingen). All samples were finally treated with streptavidin-horseradish peroxidase and stained with TSA Fluorescence/Cyanine 3 systems according to the manufacturer’s instructions (Fluorescein NEL701 A, Cyanine NEL 704 A, PerkinElmer).

### Statistics

For the animal experiments in order to determine group size necessary for adequate statistical power, power analysis employing the programme G*power was performed using preliminary data sets. Mice of the indicated genotype were assigned at random to groups. Mouse studies were performed in a blinded fashion. *P* values were calculated with a two-tailed unpaired Student’s *t*-test or for the comparison of more than two conditions with a one- or two-way ANOVA followed by Fisher LSD. *P* values of 0.05 or less were considered significant, *p ≤ 0.05, **p ≤ 0.01 and ***p ≤ 0.001.

### Data availability

The authors declare that the data supporting the findings of this study are available within the article and its supplementary information files, or are available upon reasonable requests to the authors. Expression data are available at the Gene Express Omnibus database (http://www.ncbi.nlm.nih.gov/geo/) with accession number GSE111450.

## Electronic supplementary material

Supplementary Information
