# Supplementary material for: Obesity exacerbates colitis-associated cancer via IL-6-regulated macrophage polarisation and CCL-20/CCR-6-mediated lymphocyte recruitment
Source: Nat Commun. 2018 Apr 25;9:1646. doi: 10.1038/s41467-018-03773-0 (PMC5916940; doi:10.1038/s41467-018-03773-0)
Supplement: Supplementary file 1 — Supplementary Information [file 41467_2018_3773_MOESM1_ESM.pdf]

Supplementary information

Obesity exacerbates colitis-associated cancer via IL-6-regulated macrophage polarisation and CCL-20/CCR-6-mediated lymphocyte recruitment

Wunderlich et al.,

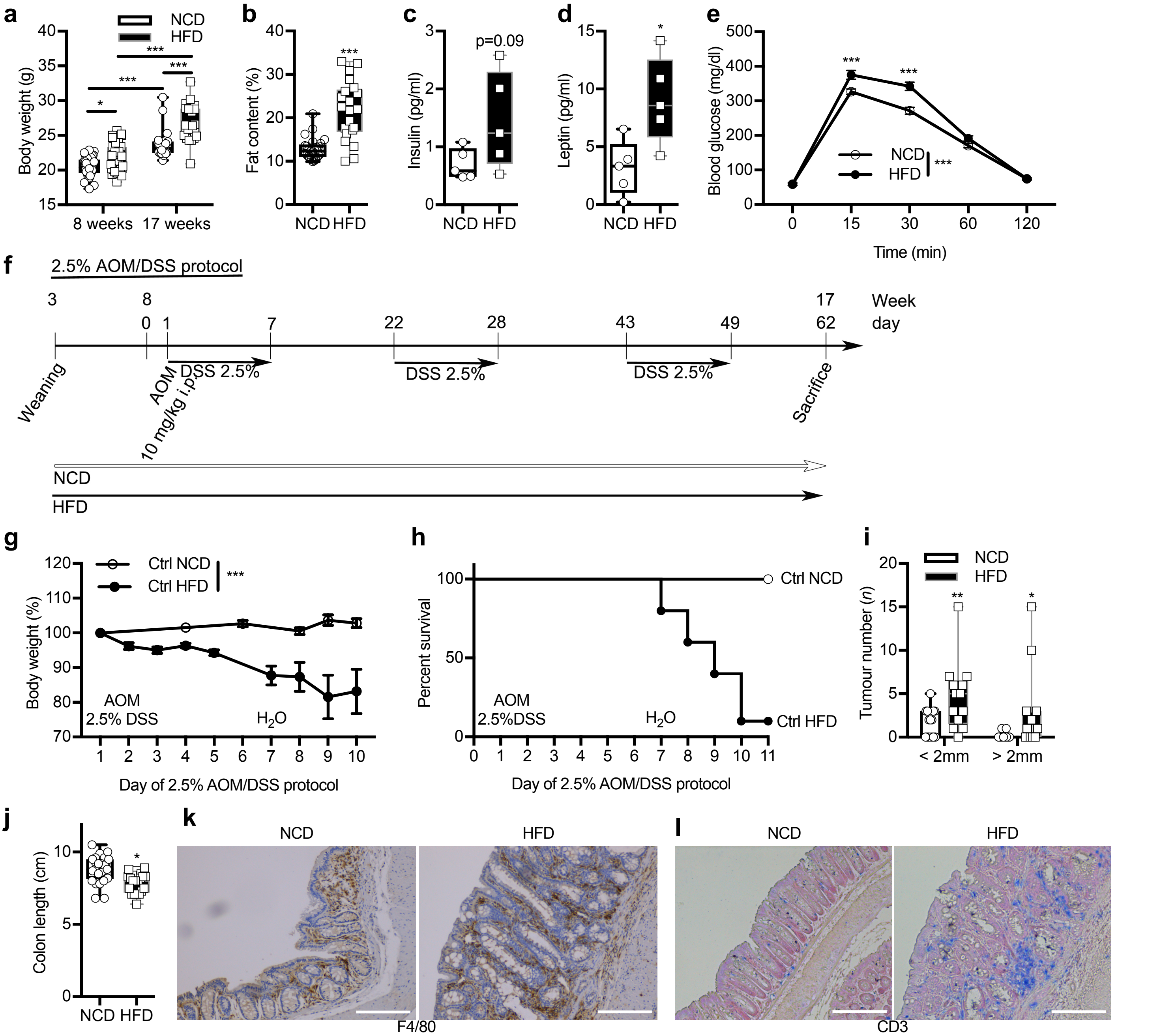

Supplementary Fig. 1 Metabolic parameters of NCD and HFD-fed C57BL/6 mice in CAC. Metabolic parameters of C57BL/6 mice exposed to NCD (white) or HFD (black) feeding: **a** body weight at 8 weeks of age (day 0) and at 17 weeks of age (day 62) after the 1.5% AOM/DSS protocol (*n*=21-41), **b** fat content at 17 weeks of age (day 62) after the 1.5% AOM/DSS protocol examined by nuclear magnetic resonance (*n*=25, *n*=21), **c** fed serum insulin levels (*n*=5), **d** fed serum leptin levels (*n*=5) as examined by ELISA, **e** glucose tolerance test of 7-8-week-old non-colitic mice (*n*=10). **f** Scheme of the 2.5% AOM/DSS protocol of mice fed a NCD or a HFD from weaning (3 weeks of age) until the end of the experiment with 17 weeks of age (day 62). 8-week-old mice were injected with 10 mg/kg azoxymethane (AOM) i.p. at day 1 of the 2.5% AOM/DSS protocol and received 2.5% dextran sodium sulfat (DSS) in the drinking water three times for 7 days from day 1-7, day 22-28 and day 43-49. Mice were killed with 17 weeks of age at day 62 for analysis of tumourigenesis. **g** Body weight loss and **h** survival curve in % of 8-10-week-old Ctrl NCD and Ctrl HFD-fed mice (*n*=9-10) from day 1 to day 10/day 11 of the 2.5% AOM/DSS protocol. **i** Tumour number <2 mm and >2 mm of 17-week-old NCD (*n*=18) and HFD-fed (*n*=13) C57BL/6 mice counted at day 62 of the 1.5% AOM/DSS protocol. **j** Colon length from rectum to caecum at day 62 of the 1.5% AOM/DSS protocol from 17-week-old mice (*n*=21-26). Representative immunohistochemical determination of **k** F4/80 (brown) and **l** CD3 (blue) expressing cells in distal colons from NCD-fed and HFD-fed 17-week-old C57BL/6 mice at day 62 of the 1.5% AOM/DSS protocol. NCD, normal chow diet; HFD, high fat died; CAC, colitis-associated colorectal cancer. Data are represented as mean ± SEM or center line: median; box limits: 1st and 3rd quartiles; whisker: maximum to minimum, *$p \leq 0.05$, **$p \leq 0.01$ and ***$p \leq 0.001$ two-tailed unpaired Student's *t*-test **b**, **c**, **d**, **i**, **j** or two-way ANOVA followed by Fisher LSD **a**, **e**, **g**. Scale bar, 100 μm (k,l).

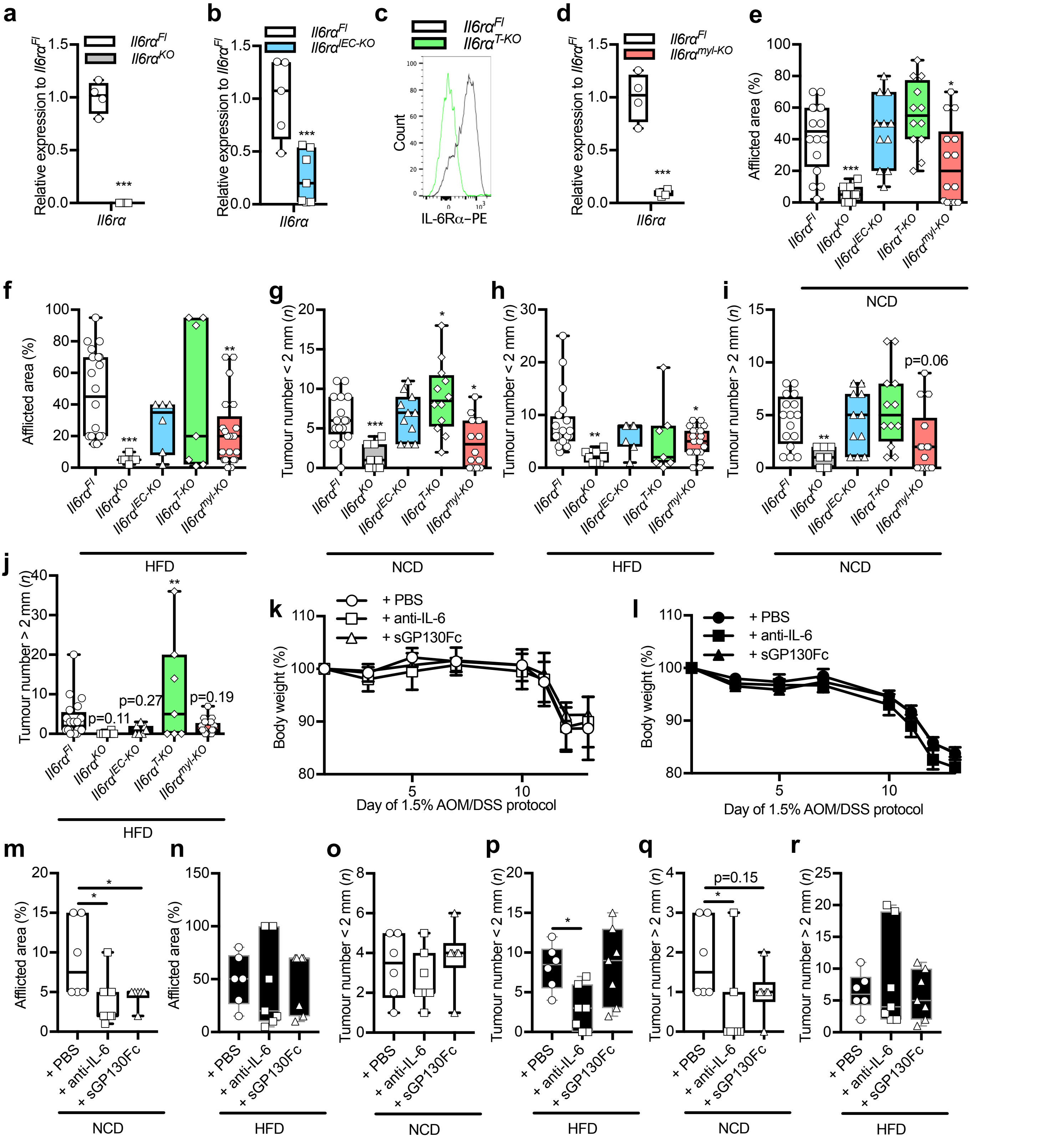

Supplementary Fig. 2 Conditional *Il6rα* inactivation. qPCR analysis of *Il6rα* gene expression in **a** control $Il6r\alpha^{Fl}$ and $Il6r\alpha^{KO}$ colons (*n*=5) and in **b** control $Il6r\alpha^{Fl}$ and intestinal epithelial-specific $Il6r\alpha^{IEC\text{-}KO}$ IECs (*n*=7); results are presented relative to $Il6r\alpha^{Fl}$. **c** FACS analysis of lymphocytes from control $Il6r\alpha^{Fl}$ and T cell-specific $Il6r\alpha^{T\text{-}KO}$ mice pregated with anti-CD3, histogram for anti-IL-6Rα expression. **d** qPCR analysis of *Il6rα* gene expression in control $Il6r\alpha^{Fl}$ and $Il6r\alpha^{myl\text{-}KO}$ BMDM (*n*=4), results are presented relative to $Il6r\alpha^{Fl}$ BMDM. Afflicted area of distal colons with tumours in 17-week-old **e** NCD (*n*=9-16) and **f** HFD-fed (*n*=7-20) $Il6r\alpha^{Fl}$, $Il6r\alpha^{KO}$, $Il6r\alpha^{IEC\text{-}KO}$, $Il6r\alpha^{T\text{-}KO}$, $Il6r\alpha^{myl\text{-}KO}$ mice at day 62 of the 1.5% AOM/DSS protocol. Tumour number <2 mm of 17-week-old **g** NCD (*n*=9-16) and **h** HFD-fed (*n*=7-20) $Il6r\alpha^{Fl}$, $Il6r\alpha^{KO}$, $Il6r\alpha^{IEC\text{-}KO}$, $Il6r\alpha^{T\text{-}KO}$, $Il6r\alpha^{myl\text{-}KO}$ mice counted at day 62 of the 1.5% AOM/DSS protocol. Tumour number >2 mm of 17-week-old **i** NCD (*n*=9-16) and **j** HFD-fed (*n*=7-20) $Il6r\alpha^{Fl}$, $Il6r\alpha^{KO}$, $Il6r\alpha^{IEC\text{-}KO}$, $Il6r\alpha^{T\text{-}KO}$, $Il6r\alpha^{myl\text{-}KO}$ mice counted at day 62 of the 1.5% AOM/DSS protocol. Body weight loss in % of **k** NCD (*n*=6-7) or **l** HFD-fed (*n*=6-7) C57BL/6 mice from day 1 to day 13 of the 1.5% AOM/DSS protocol. Afflicted area of distal colons with tumours in 17-week-old **m** NCD (*n*=6-7) and **n** HFD-fed (*n*=6-7) C57BL/6 mice at day 62 injected with either PBS, 500 µg anti-IL-6 antibody or 150 µg sGP130Fc at day 3 of the 1.5% AOM/DSS protocol. Tumour number <2 mm of 17-week-old **o** NCD (*n*=6-7) and **p** HFD-fed (*n*=6-7) C57BL/6 mice at day 62 injected with either PBS, 500 µg anti-IL-6 antibody or 150 µg sGP130Fc at day 3 of the 1.5% AOM/DSS protocol. Tumour number >2 mm of 17-week-old **q** NCD (*n*=6-7) and **r** HFD-fed (*n*=6-7) C57BL/6 mice at day 62 injected with either PBS, 500 µg anti-IL-6 antibody or 150 µg sGP130Fc at day 3 of the 1.5% AOM/DSS protocol. AOM, azoxymethane; DSS, dextran sodium sulphate; NCD, normal chow diet; HFD, high fat died; BMDM, bone marrow derived macrophages; IEC, intestinal epithelial cells. Data are represented as mean ± SEM or center line: median; box limits: 1st and 3rd quartiles; whisker: maximum to minimum, *$p\leq0.05$, **$p\leq0.01$ and ***$p\leq0.001$ two-tailed unpaired Student's *t*-test **a** ,**b**, **d**, one-way **e**, **f**, **g**, **h**, **i**, **j**, **m**, **n**, **o**, **p**, **q**, **r** or two-way ANOVA followed by Fisher LSD **k**, **l**.

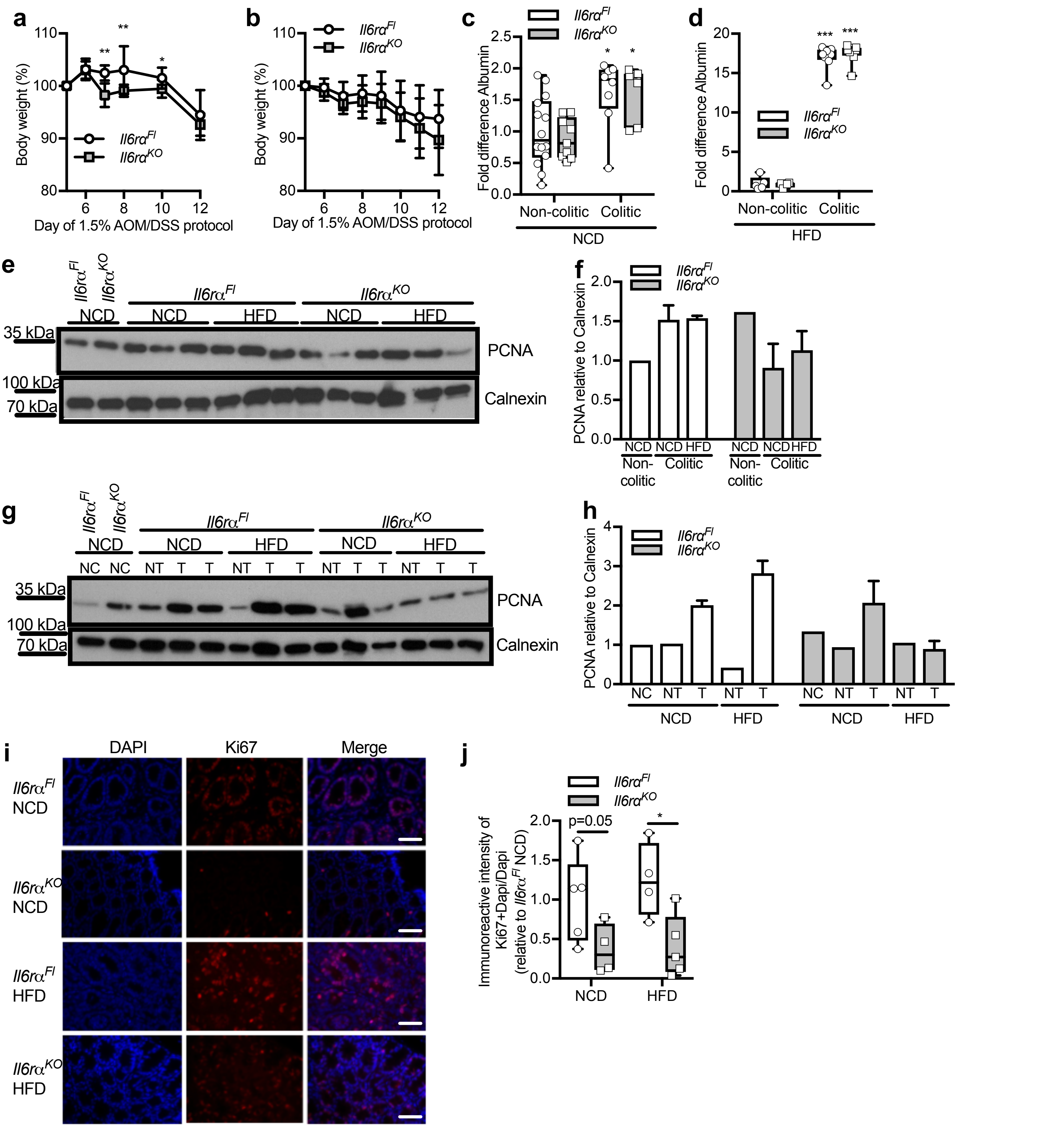

Supplementary Fig. 3 Characteristics of *Il6rα*^{Fl} and *Il6rα*^{KO} mice in CAC. Body weight loss in % of **a** NCD (*n*=10) or **b** HFD-fed (*n*=6-8) *Il6rα*^{Fl} and *Il6rα*^{KO} mice from day 5 to day 12 of the 1.5% AOM/DSS protocol. Normalised albumin concentration in faeces of non-colitic 8- and colitic 10-week-old *Il6rα*^{Fl} and *Il6rα*^{KO} mice at day 0 and day 13 of the 1.5% AOM/DSS protocol fed a **c** NCD (*n*=7-14) or a **d** HFD (*n*=4-8); results are presented relative to *Il6rα*^{Fl} non-colitic NCD or HFD respectively. **e** Immunoblot analysis of PCNA and Calnexin (loading control) in non-colitic (first two columns) at day 0 and colitic distal colon tissue at day 13 of the 1.5% AOM/DSS protocol from *Il6rα*^{Fl} and *Il6rα*^{KO} mice fed a NCD or HFD and its **f** quantification. **g** Immunoblot analysis of PCNA and Calnexin (loading control) in non-colitic (NC), non-tumour tissue (NT) and tumour tissue (T) of colons at day 62 of the 1.5% AOM/DSS protocol from 17-week-old *Il6rα*^{Fl} and *Il6rα*^{KO} mice fed a NCD or HFD and its **h** quantification. **i** Representative immunofluorescent staining of Ki67 proliferating IECs in colons of 17-week-old *Il6rα*^{Fl} and *Il6rα*^{KO} mice at day 62 of the 1.5% AOM/DSS protocol using Ki67 antibody (red) counterstained by DAPI (blue) and its **j** quantification, immunoreactive intensity of Ki67 and DAPI double positive cells set relative to all DAPI positive cells (*n*=4-5). AOM, azoxymethane; DSS, dextran sodium sulphate; NCD, normal chow diet; HFD, high fat died; CAC, colitis-associated colorectal cancer; PCNA, proliferating cell nuclear antigen; IEC, intestinal epithelial cells. Data are represented as mean ± SEM or center line: median; box limits: 1st and 3rd quartiles; whisker: maximum to minimum, *$p \leq 0.05$, **$p \leq 0.01$ and ***$p \leq 0.001$ two-way ANOVA followed by Fisher LSD **a**, **b**, **c**, **d**, **j**. Scale bar, 50 μm **i**.

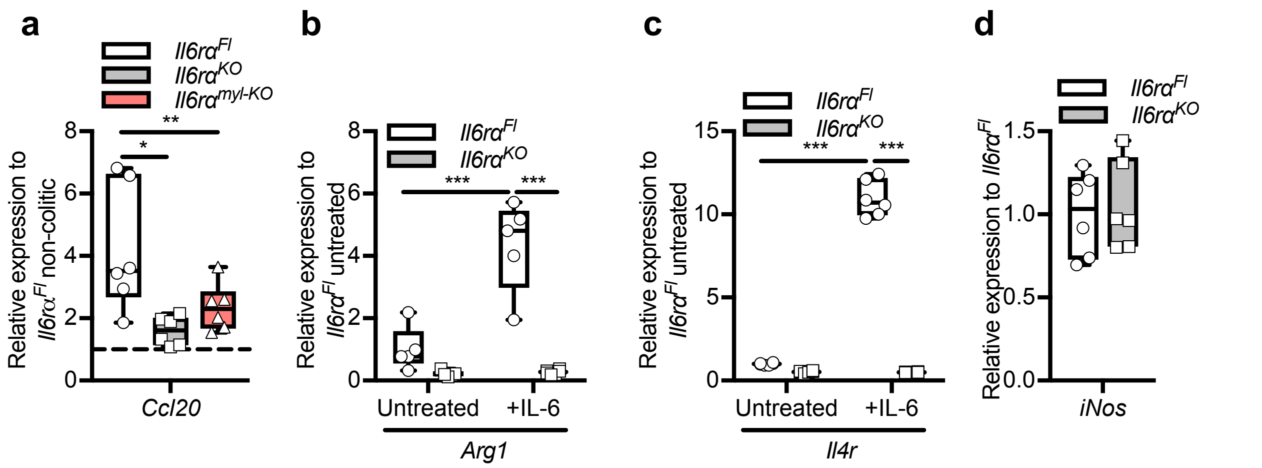

Supplementary Fig. 4 IL-6 signalling in macrophage promotes M2 polarisation. **a** qPCR analysis of *Ccl20* gene expression in isolated IECs from colitic colons from 10-week-old *Il6r*α*^Fl^* (*n*=6), *Il6r*α*^KO^* (*n*=6) and *Il6r*α*^myl-KO^* (*n*=6) NCD-fed mice at day 13 of the 1.5% AOM/DSS protocol, results are presented relative to non-colitic *Il6r*α*^Fl^* IECs. qPCR analysis of **b** *Arg1* and **c** *Il4r* gene expression in control *Il6r*α*^Fl^* versus *Il6r*α*^KO^* M2 polarised and IL-6 treated BMDM (*n*=6) for 8 h, results are presented relative to *Il6r*α*^Fl^* BMDM. **d** qPCR analysis of *iNos* gene expression in control *Il6r*α*^Fl^* versus *Il6r*α*^KO^* M1 polarised BMDM (*n*=6), results are presented relative to control BMDM. AOM, azoxymethane; DSS, dextran sodium sulphate; NCD, normal chow diet; IEC, intestinal epithelial cells; BMDM, bone marrow derived macrophages. Center line: median; box limits: 1st and 3rd quartiles; whisker: maximum to minimum, *$p \leq 0.05$, **$p \leq 0.01$ and ***$p \leq 0.001$ two-tailed unpaired Student's *t*-test **d** or one-way **a** or two-way ANOVA followed by Fisher LSD **b**, **c**.

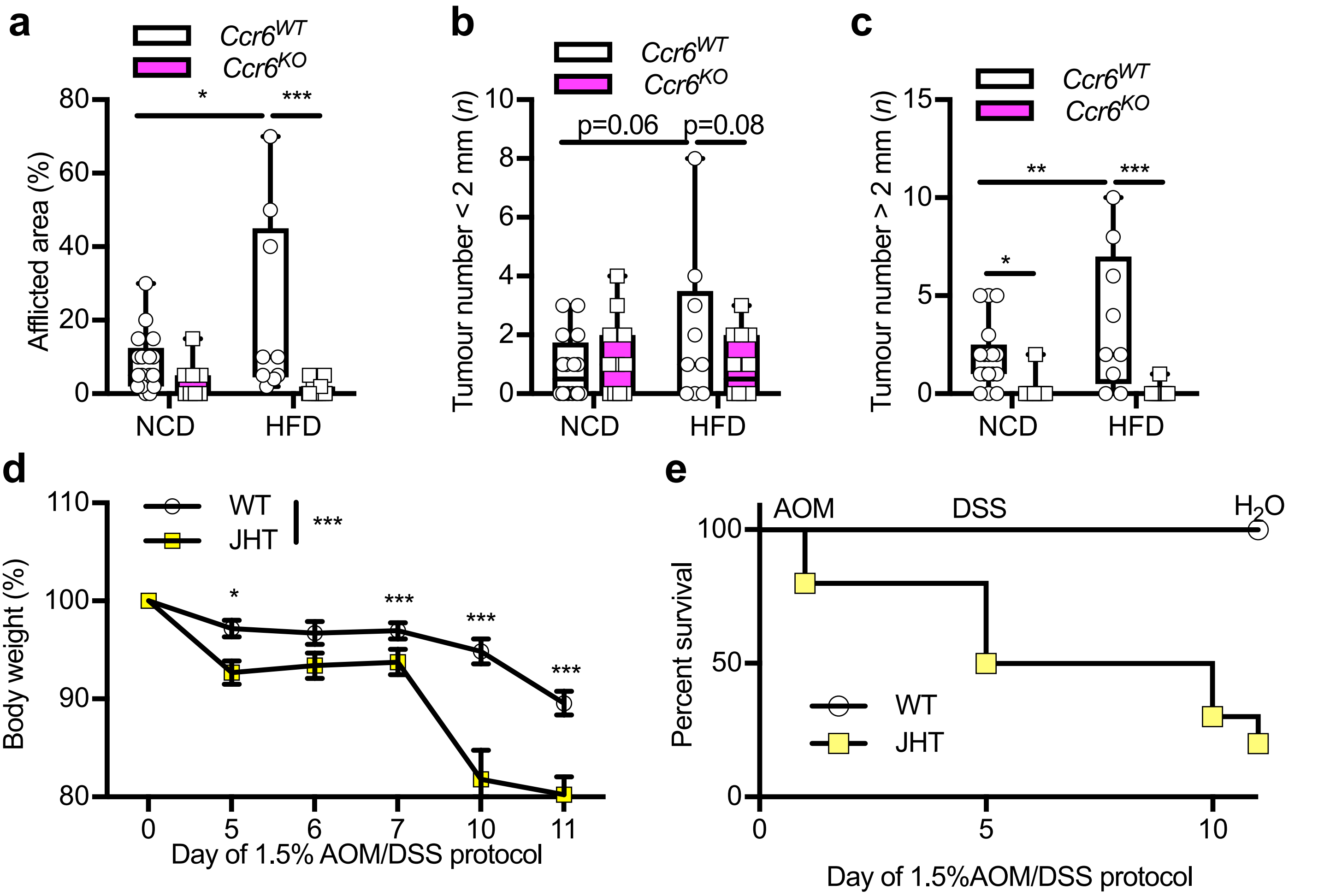

Supplementary Fig. 5 Tumour size of *Ccr6*^{WT} and *Ccr6*^{KO} mice and obese JHT mice in CAC. **a** Afflicted area of distal colons with tumours in 17-week-old NCD (*n*=10-17) and HFD-fed (*n*=9-11) *Ccr6*^{WT} and *Ccr6*^{KO} mice at day 62 of the 1.5% AOM/DSS protocol. **b** Tumour number <2 mm of 17-week-old NCD (*n*=10-17) and HFD-fed (n=9-11) *Ccr6*^{WT} and *Ccr6*^{KO} mice at day 62 of the 1.5% AOM/DSS protocol. **c** Tumour number >2 mm of 17-week-old NCD (*n*=10-17) and HFD-fed (*n*=9-11) *Ccr6*^{WT} and *Ccr6*^{KO} mice at day 62 of the 1.5% AOM/DSS protocol. **d** Body weight loss in % of HFD-fed WT (*n*=8) and JHT (*n*=9) mice at day 0 to day 11 of the 1.5% AOM/DSS protocol. **e** Kaplan-Meier survival curve of HFD-fed WT (*n*=8) and JHT (*n*=9) mice at day 0 to day 11 of the 1.5% AOM/DSS protocol. AOM, azoxymethane; DSS, dextran sodium sulphate; NCD, normal chow diet; HFD, high fat died; CAC, colitis-associated colorectal cancer; JHT, targeted deletion of the JH locus. Data are represented as mean ± SEM or center line: median; box limits: 1st and 3rd quartiles; whisker: maximum to minimum, *p≤0.05, **p≤0.01 and ***p≤0.001 two-way ANOVA followed by Fisher LSD **a**, **b**, **c**, **d**.

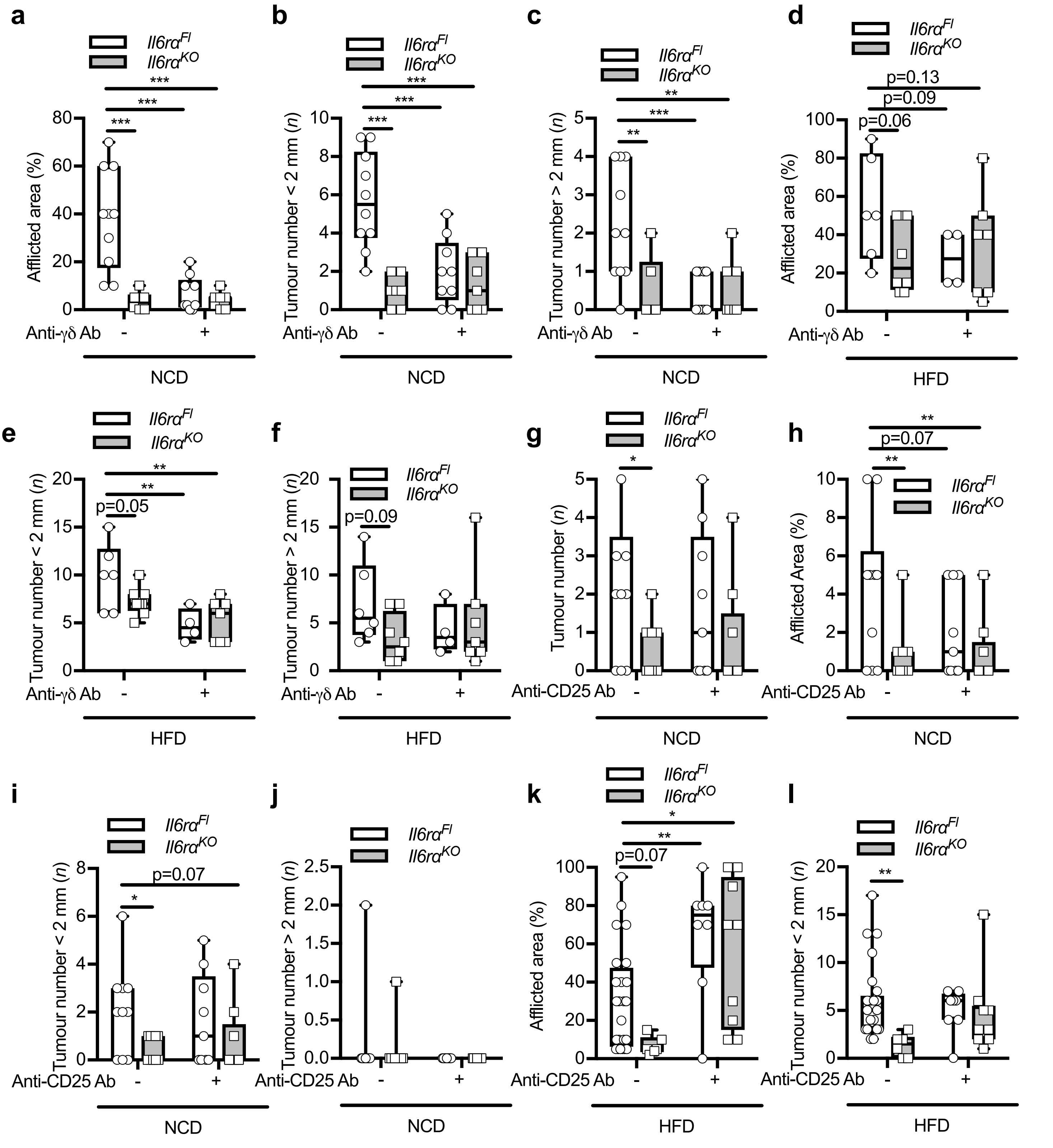

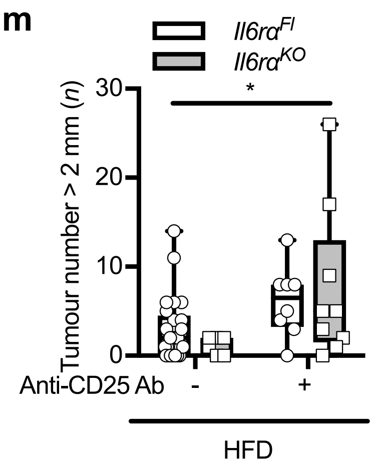

Supplementary Fig. 6 Tumour sizes of anti-γδ and anti-CD25 antibody treated $Il6r\alpha^{Fl}$ and $Il6r\alpha^{KO}$ mice. **a** Afflicted area of distal colons with tumours, **b** tumour number <2 mm and **c** tumour number >2 mm in 17-week-old NCD-fed (*n*=6-10) $Il6r\alpha^{Fl}$ and $Il6r\alpha^{KO}$ mice at day 62 injected either with PBS or 200 µg anti-γδ antibody at day 3 of the 1.5% AOM/DSS protocol. **d** Afflicted area of distal colons with tumours, **e** tumour number <2 mm and **f** tumour number >2 mm in 17-week-old HFD-fed (*n*=4-8) $Il6r\alpha^{Fl}$ and $Il6r\alpha^{KO}$ mice at day 62 injected either with PBS or 200 µg anti-γδ antibody at day 3 of the 1.5% AOM/DSS protocol. **g** Tumour number, **h** afflicted area of distal colons with tumours, **i** tumour number <2 mm and **j** tumour number >2 mm in 17-week-old NCD-fed $Il6r\alpha^{Fl}$ and $Il6r\alpha^{KO}$ mice (*n*=9-10) at day 62 injected with either PBS or 1 mg anti-CD25 antibody at day 3 of the 1.5% AOM/DSS protocol. **k** Afflicted area of distal colons with tumours, **l** tumour number <2 mm and **m** tumour number >2 mm in 17-week-old HFD-fed (*n*=6-24) $Il6r\alpha^{Fl}$ and $Il6r\alpha^{KO}$ mice at day 62 injected either with PBS or 1 mg anti-CD25 antibody at day 3 of the 1.5% AOM/DSS protocol. AOM, azoxymethane; DSS, dextran sodium sulphate; NCD, normal chow diet; HFD, high fat died; Ab, Antibody. Center line: median; box limits: 1st and 3rd quartiles; whisker: maximum to minimum, $*p \leq 0.05$, $**p \leq 0.01$ and $***p \leq 0.001$ two-way ANOVA followed by Fisher LSD **a**, **b**, **c**, **d**, **e**, **f**, **g**, **h**, **i**, **j**, **k**, **l**, **m**.

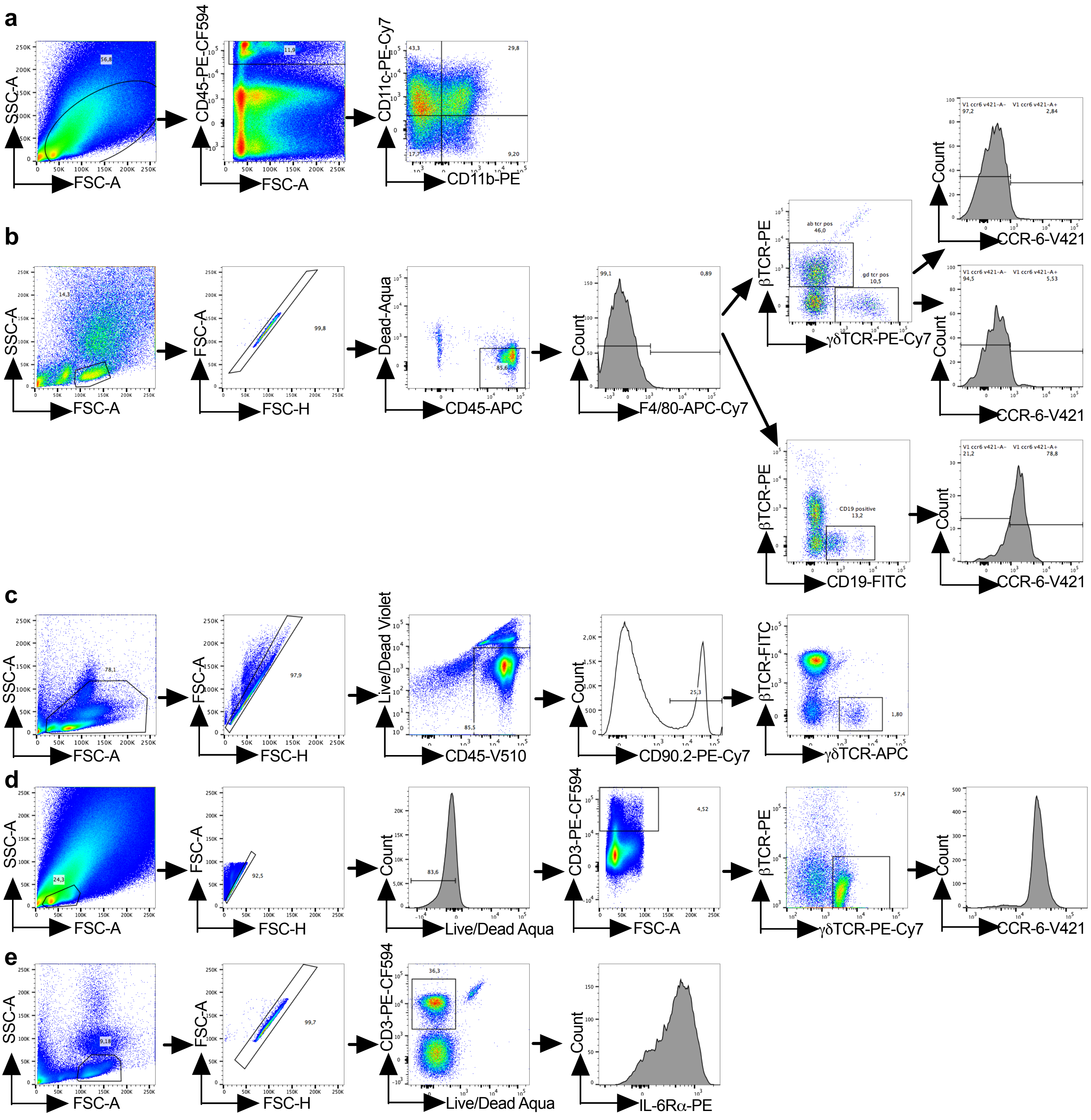

Supplementary Fig. 7 Representative FACS gating strategies**.** Gating strategies for **a** Fig. 2h+i; **b** Fig. 4f,g,h; **c** Fig. 7a; **d** Fig. 7b; **e** Supplementary Fig. 2c.

a

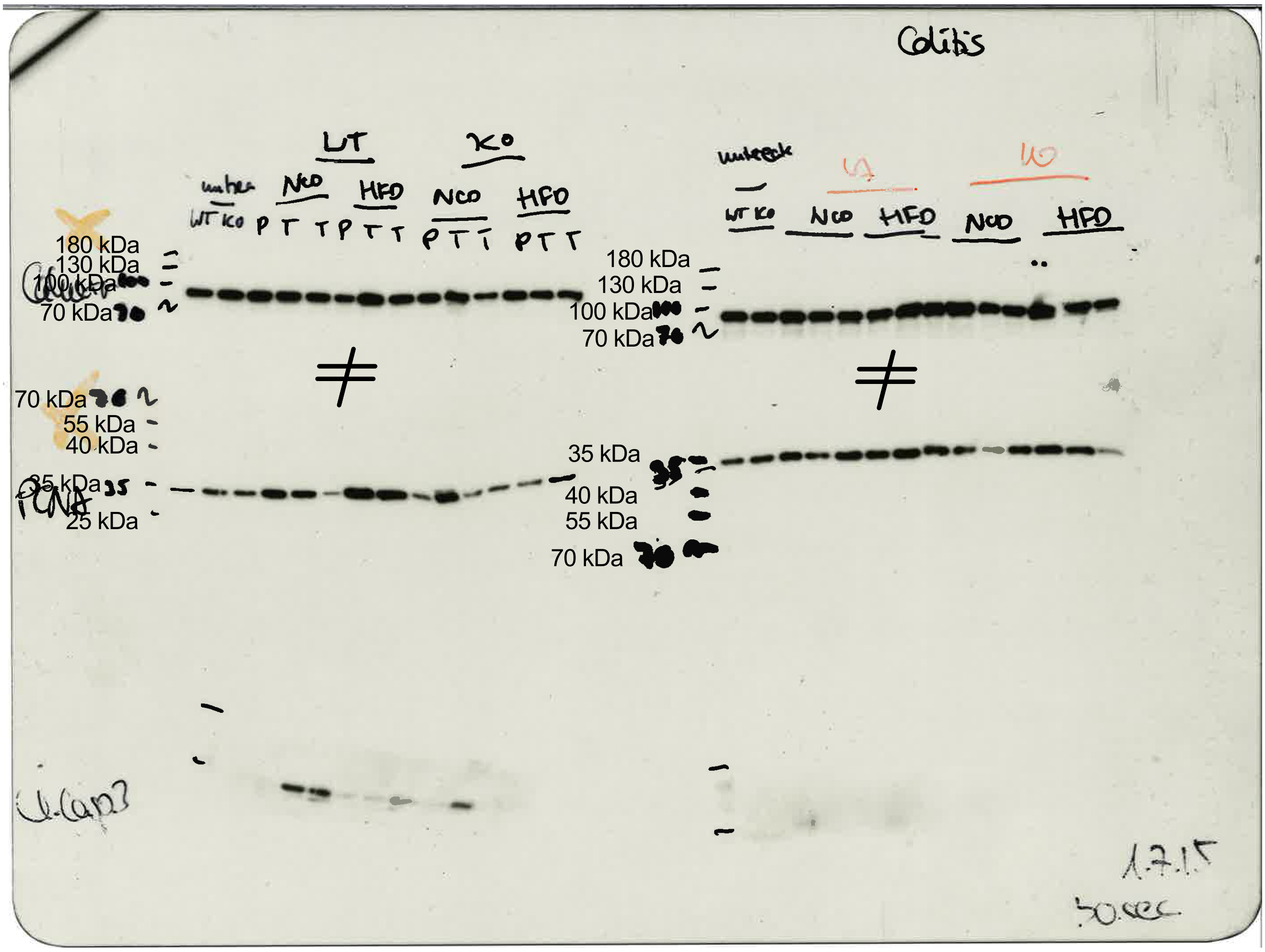

Supplementary Fig. 8 Original data of immunoblots. Immunoblot data corresponding to Supplemetary Fig. 3e+g. Immunoblots were cut at 70 kDa, the upper part was incubated with anti-Calnexin antibody, the lower part with anti-proliferating cell nuclear antigen (PCNA) antibody.

Supplementary Table 1 List of downregulated genes in IL-6Rα-deficent tumours

!"#	$%"&'(')*+,	-'.'*/((0-.1'.)	23*)45,6)*)45,7"	$8*)45,6)*)45,7"
9	9:;;<99=	>&?@AA@B&0CB0)0.@,	DEFGH;G=9IEH	:F:==;9==;=
=	9:<EIH:H	J.KE@AA@J!LF@ME@(1/NN@3K)"$N/(103@O/(("30/)'?@60)P@J"@$%")'0.Q	DEF9E;;:RI<I	:F::G;R=:;9
E	9:EG9H:G	+N==%/=@AA@0.)'%N'B70.@==@%'3'$)"%F@/N$P/@=	D=FR;ER<=<<E	:F:::E<I<;;
;	9:ER<:RE	S/&%$@AA@-/11/D/10."&B)K%03@/30?@OSLTLQ@L@%'3'$)"%F@$0	D=FGIH9=9;R=	:F::9=;I9I9
<	9:<G99G=	UK$=/==@AA@3K)"3P%"1'@V;<:F@2/10NK@=F@(B&2/10NK@/F@$"NK$'$)0?'@==@	D=FGER;9RE;=	:F:==HGEE9G
G	9:;:IERI	S1I9=:@AA@$%'?03)'?@-'.'@I9=:	D=FG=R=GEG;=	:F:=;G<9HHH
I	9:E;<:IH	WP?39/@AA@W4@?"1/0.@3".)/0.0.-@9L	D=FEIG9:9RE=	:F:=;;E9E;I
R	9:<GIRGE	U?9H@AA@U,9H@/.)0-'.	D=FEG<9;I=;	:F:::;RG9E9
H	9:E;RH:G	S1G:RG@AA@$%'?03)'?@-'.'@G:RG	D=F=<;R9G;<E	:F:::;I;GRI
9:	9:;<G=;R	!X*9H;=GR@AA@Y.'3B)=@AA@*.'@3B)@?"1/0.F@2/10NK@1'1&'%@=	D=F=;RE<::;G	:F:9GI;EG=R
99	9:E;IRRR	U3N=:@AA@3P'1"70.'@OUDU@1")02Q@N0-/.?@=:	D=F==;IE;I;;	:F::<G=RE:9
9=	9:<9=GGH	V/Z<@AA@$/0%'?@&"Z@-'.'@<	D=F=:=;::=E9	:F::99<EE=;
9E	9:;=I=H:	4"Z3R@AA@P"1'"@&"Z@UR	D=F9:R9RII9R	:F::I=<I;=9
9;	9:;IEEHH	V%-=@AA@$%")'"-NK3/.@=F@&".'@1/%%"6	D=F:IR;G=;=<	:F:9:=;;E=;
9<	9:<GR:=;	U"%"9/@AA@3"%".0.F@/3)0.@&0.?0.-@$%")'0.@9L	D=F:I:R;:R=I	:F:::9G;GHR
9G	9:<G=R9=	($0&@AA@($0DT@)%/.(3%0$)0".@2/3)"%@O($0D9AV>#9@%'N/)'?Q	D=F:G:<IHE=	:F:::<;EIRG
9I	9:EH=9;=	U?IH&@AA@U,IHT@/.)0-'.	D=F:<;:R9EI	:F:::GIRIGR
9R	9:EHGG<=	4($/=@AA@P'/)@(P"37@$%")'0.@=	D=F:;<I9=EHI	:F::9<=I=H
9H	9:;=99::	!'21@AA@.'B%"20N/1'.)F@1'?0B1@$"NK$'$)0?'	D=F:;<:EGG:<	:F::H:GGE;=
=:	9:<IGI<I	53'%=/@AA@53@%'3'$)"%F@+-\F@N"6@/220.0)K@++F@/N$P/@$"NK$'$)0?'	D=F:;9EGER=I	:F:::;I=9:IH
=9	9:<GE9IR	U?EI@AA@U,EI@/.)0-'.	D=F:EER<;II=	:F::EIE9;;9
==	9:;ER;;<	WNPNG@AA@7'N3PDN07'@G@O,%"("$P0N/Q	D=F:9EG==G=	:F::;=<R9EH
=E	9:;<<:<;	V3?P&E@AA@$%")"3/?P'%0.@&')/@E	D9FHHIHII:E;	:F::E9;<;=
=;	9:;9=EE<	+(N9@AA@+[]9@)%/.(3%0$)0".@2/3)"%F@]+XAP"1'"?"1/0.	D9FHHG:;EIEI	:F:99R=<IIG
=<	9:;:EH;E	40()9P=&1@AA@P0()".'@3NB()'%@9@F@4=&1	D9FHR=IIEGG=	:F::=H<ERH9
=G	9:;IGE=9	V%.?@AA@$%0".@$%")'0.@?B&N')	D9FHI:E=::<H	:F::G9;H9R
=I	9:;;9GEE	U3%G@AA@3P'1"70.'@OUDU@1")02Q@%'3'$)"%@G	D9FH<IR;HGIR	:F::<:=<:;=
=R	9:<;<9R=	S1;<H@AA@$%'?03)'?@-'.'@;<H	D9FH;R;=I9G=	:F:9H9E;;=<
=H	9:;IRGEE	X1$H@AA@1/)%0Z@1')/NN"$'$)0?/('@H	D9FH;=;I<R<G	:F::II9:E<G
[illegible]	[illegible]	[illegible]	[illegible]	[illegible]
